# Management of Rhythm and Conduction Disorders in Cardiac Amyloidosis: A French Nationwide Delphi Study

**DOI:** 10.1016/j.jacadv.2025.101604

**Published:** 2025-02-15

**Authors:** Charles Guenancia, Benoit Lequeux, Walid Amara, Otilia Buiciuc, Thibaud Damy, Pascal Defaye, Alexandre Duparc, Jean-Christophe Eicher, Rodrigue Garcia, Vincent Galand, Olivier Lairez, Nicolas Lellouche, Silvia Oghina

**Affiliations:** a Cardiology Department, Dijon University Hospital, Dijon, France; b Laboratory of Cerebro-Vascular Pathophysiology and Epidemiology (PEC2) EA 7460, University of Bourgogne, Dijon, France; c Department of Cardiology, University Hospital of Poitiers, Poitiers, France; d Cardiology Department, Le Raincy Montfermeil Hospital, Montfermeil, France; e Cardiac Arrhythmia Service, Amiens-Picardie University Hospital, Amiens, France; f French Referral Centre for Cardiac Amyloidosis, GRC Amyloid Research Institute, Amyloidosis Mondor Network, and DHU A-TVB, Henri Mondor Teaching Hospital, APHP, Creteil, France; g Department of Cardiology, Henri Mondor Teaching Hospital, Creteil, France; h Cardiology Department, Grenoble University Hospital and INSERM LRB Grenoble Alpes University, Grenoble, France; i Department of Electrophysiology and Pacing, Toulouse University Hospital, Toulouse, France; j Centre d'Investigation Clinique 1402, University Hospital of Poitiers, Poitiers, France; k Interventional Cardiology Department, Saint Joseph Clinic, Trélazé, France; l Department of Cardiology and Cardiac Imaging Center, University Hospital of Rangueil, Toulouse, France

**Keywords:** atrial fibrillation, cardiac amyloidosis, Delphi method, implantable cardioverter defibrillator, pacemaker, ventricular arrhythmias

## Abstract

**Background:**

Cardiac amyloidosis (CA) is an increasingly recognized cardiomyopathy with an associated risk of arrhythmias and conduction disorders; however, managing arrhythmias and conductive disorders remains largely undefined.

**Objectives:**

This study aims to gather French expert experience on current practices and treatment strategies for managing arrhythmias and conduction disorders in CA. The main areas of interest included atrial fibrillation (AF) management, anticoagulation therapy, and criteria for implanting cardiac rhythm devices.

**Methods:**

A modified Delphi method was employed, involving a panel of 56 cardiologists and electrophysiologists specializing in CA. The panel evaluated 248 statements over 2 rounds. Consensus was defined as agreement from at least 66.7% of the panel, with strong consensus requiring more than 50% complete agreement.

**Results:**

Consensus was achieved on 177 out of 248 statements across 2 rounds (71%). Key agreements included 1) the necessity for regular Holter monitoring and anticoagulation therapy in high-risk scenarios; 2) a rhythm control management strategy, including the use of amiodarone and AF ablation, particularly in the early stages of the disease; and 3) the use of cardiac devices for advanced conduction disorders, with decisions influenced by disease staging and left ventricular ejection fraction.

**Conclusions:**

Approximately 70% of the proposed statements achieved agreement among the experts, reflecting reasonable alignment on anticoagulation therapy, AF management, and implantable cardiac devices. However, the study also highlights the need for personalized, multidisciplinary management of arrhythmias and conduction disorders in CA and emphasizes the need for future research to develop evidence-based guidelines.

Cardiac amyloidosis (CA) is an increasingly recognized form of cardiomyopathy stemming from systemic dysfunctions. It is characterized by the accumulation of insoluble amyloid fibrils, notably in the myocardial extracellular matrix.^1^^,^^2^ This systemic condition primarily manifests in 2 major subtypes: light-chain amyloidosis (AL) and transthyretin amyloidosis (ATTR), with the latter further subdivided into wild-type (ATTRwt) and hereditary (ATTRv) forms.^2^, ^3^, ^4^, ^5^ Each subtype presents distinctive clinical features with an important impact on health-related quality of life associated with high mortality of 30% to 40% at 3.5 years for ATTR and around 50% for AL at 5-year follow-up.^6^, ^7^, ^8^

The accumulation of amyloid fibrils in all cardiac structures clinically leads to increased wall thickness and stiffness, causing heart failure, conductive disorders, arrhythmias, and cardioembolic events.^4^^,^^9^^,^^10^ In advanced stages, systolic dysfunction may also occur. The disruptions of the normal cardiac architecture are more pronounced in ATTRv, where conduction system involvement is frequent and often necessitates pacemaker (PM) implantation. Atrial fibrillation (AF) is a prevalent arrhythmia in ATTR, particularly among patients with ATTRwt, exacerbating diastolic dysfunction and elevating the risk of intracardiac thrombus formation.^11^^,^^12^

Despite the importance of managing conduction and rhythm abnormalities in CA, specific guidelines are lacking. Physicians often rely on broader international guidelines, which may not fully address real-world CA management in clinical practice.^13^^,^^14^ Recent electrophysiological guidelines highlight key areas needing further evidence or expert consensus,^15^ including anticoagulation for patients without AF, the efficacy of beta-blockers and identifying patients who could benefit from their use, criteria for prophylactic PM use, and determining which subgroups would benefit from implantable cardioverter-defibrillators (ICD) and cardiac resynchronization therapy (CRT).

In the absence of large-scale studies or definitive guidelines, the Delphi method used in this study helps achieve agreement among experts. A group of French CA specialists and electrophysiologists compiled current clinical practices in France, focusing on anticoagulation protocols for patients in sinus rhythm, AF management, and criteria for PM and ICD use.^10^^,^^14^, ^15^, ^16^ This study highlights areas of agreement and identifies contested management approaches, while also guiding priorities for future clinical research.

## Materials and methods

This study used a modified electronic Delphi method over 2 rounds between November 2023 and April 2024. The Delphi method is a systematic and interactive way to gather and refine opinions from a panel of independent experts, particularly when data are limited, or complex issues need resolution. Conducted via an electronic platform, this approach is considered modified, as it enabled more efficient data collection, analysis, and feedback. This method relies on 4 key principles: participant anonymity, controlled feedback, iterative rounds, and statistical aggregation of responses.

### Panel of participants and statement formulation

A scientific committee of 13 cardiologists specialized in CA management and cardiac electrophysiologists with expertise in CA rhythm disorders was established to guide the study. Among them, 3 members, designated as scientific coordinators, formulated 248 statements in French based on their experiences and a literature review, covering topics such as anticoagulant use, AF and flutter management, and cardiac device implantation in AL and ATTR separately. The statements were reviewed and approved by the scientific committee. Each committee member nominated approximately 10 French CA experts based on their clinical experience, resulting in 71 identified experts for the Delphi process, excluding the scientific coordinators. The committee ensured geographical representation across the French territory.

### Delphi process

Two rounds of the Delphi survey were conducted electronically via the General Data Protection Regulation-compliant Panelabs platform. Panelists anonymously rated their agreement with statements on a 4-point Likert scale (1 = strongly disagree, 2 = disagree, 3 = agree, 4 = strongly agree). In the Tables, the percentages indicate the proportion of the panel who agree or disagree with each statement, with responses classified as positive (scores 3-4) or negative (scores 1-2). Consensus was defined as 66.7% agreement or disagreement, while strong consensus required over 50% full agreement or disagreement.

Statements lacking convergence in the first round were revised, if necessary, by the scientific committee during a virtual meeting in February 2024. The updated statements and first-round results were then shared for a second round of voting. A nonmandatory comment box was included in the second round to gather experts’ explanations. Statements that failed to reach consensus after the second round were classified as nonconsensual. Final analyses were presented to the scientific committee in April 2024.

In the Tables, green cells show statements with positive convergence, while dark green cells highlight strong positive convergence (≥50% strongly agree). Red cells denote negative convergence, and dark red cells indicate strong negative convergence (≥50% strongly disagree). White cells represent statements that did not reach convergence after 2 rounds.

## Results

### Voting process overview

**Figure 1:**
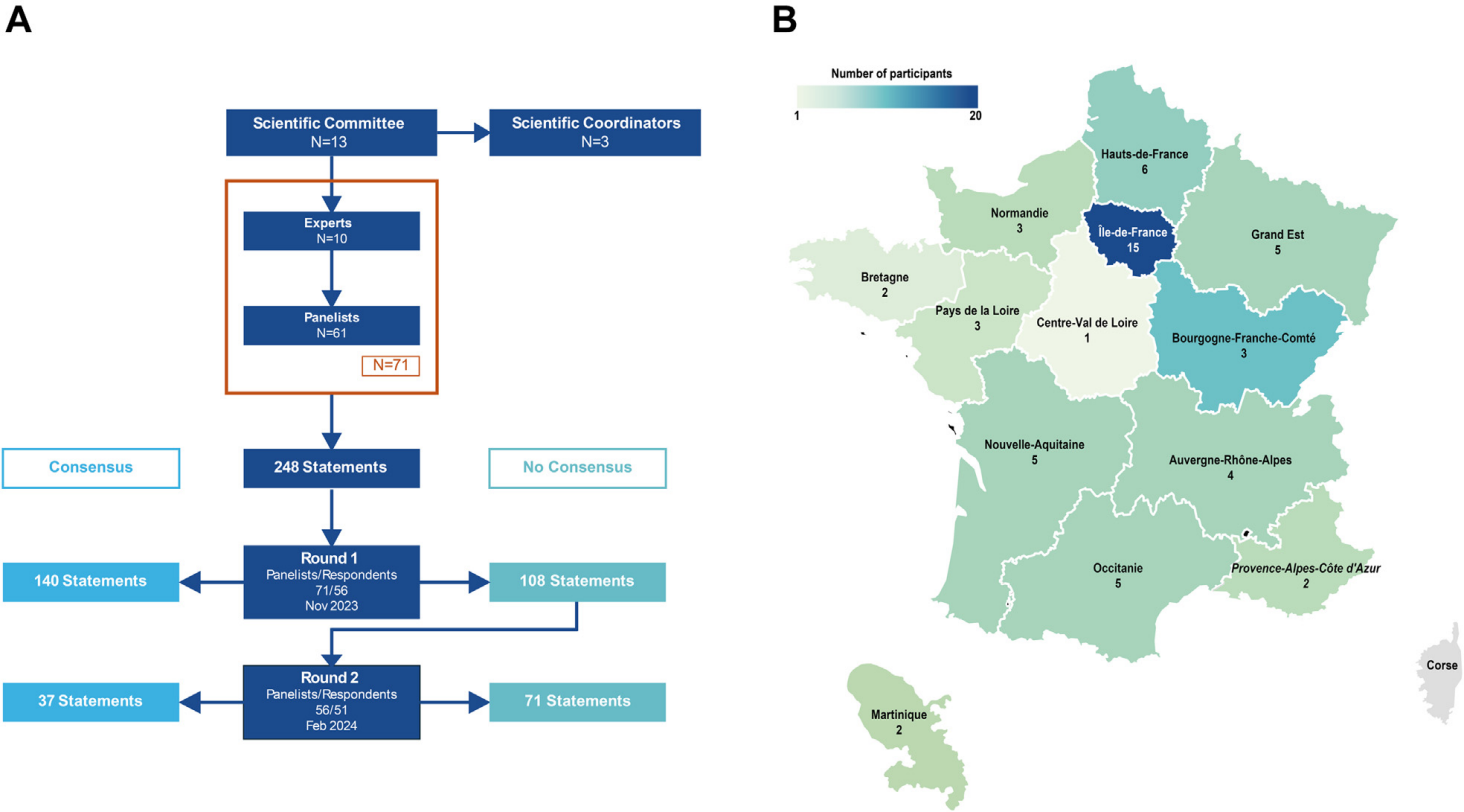
Delphi Study Process, Consensus Results, and Respondent Distribution Across France (A) The study was overseen by a 13-member scientific committee, including 3 nonvoting coordinators and 10 experts. A total of 56 panelists evaluated 248 statements. Consensus was reached on 140 statements in round 1, with an additional 37 statements achieving consensus in round 2. (B) A map illustrates the geographical distribution of experts across France (Datawrapper).

The voting process in this study evaluated 248 statements addressing anticoagulant use, AF/flutter management, and cardiac device implantation. Consensus was reached for 140 statements in the first round and 37 additional statements in the second round, leaving 71 statements without consensus (Figure 1). Expert comments were considered in the discussion. Amyloidosis severity and progression were classified using the National Amyloidosis Centre (NAC) and European staging for ATTR and AL, respectively.^17^^,^^18^ In the first round, 56 out of 71 experts participated, with a response rate of 80%. Among these, 17 were electrophysiologists (30%). Eight participants did not complete the AL amyloidosis section due to lack of expertise. In the second round, 51 experts responded, reflecting a retention rate of 89% which indicates the panel's high level of engagement with the topic. This included 17 electrophysiologists (33%), with 5 participants again not completing the AL section. Respondent distribution was geographically homogeneous and reflected medical demographics (Figure 1).

### Management of AF and flutter

Management strategies for AF and flutter showed consistency across amyloidosis subtypes. For a first episode of AF, a rhythm control strategy was preferred regardless of symptom presence. Disease severity, as assessed by NAC or European staging, did not influence management intensity in both amyloidosis subtypes (Table 1). Within rhythm control strategies, the panel primarily suggested the use of AF ablation in patients with lower staging scores, while amiodarone was endorsed across all stages. Flecainide, sotalol, and repeated electrical cardioversion (EC) were not advised in any scenario. Systematic atrial imaging was strongly suggested to detect intracardiac thrombi, even in anticoagulated patients. For typical atrial flutter, the panel strongly proposed ablation as the first-line approach.

**Table 1:** Management of Atrial Fibrillation and Flutter in Patients With ATTRv, ATTRwt, or AL

	ATTRv or ATTRwt		AL	
	Rhythm Control	Rate Control	Rhythm Control	Rate Control
Managements strategies				
First episode of symptomatic AF	96%	14%	98%	21%
First episode of asymptomatic AF	84%	32%	81%	29%
After recurrence of symptomatic EC, on amiodarone	59%	67%	70%	81%
After recurrence of asymptomatic EC, on amiodarone	21%	79%	26%	81%
If higher NAC stage, management will be more intensive	31%			
If higher European staging, management will be more intensive			36%	

		**If Higher NAC Stage**		**If Higher European Staging**

Treatment options for rate control				
Beta-blockers in progressive doses	63%	31%	63%	30%
Digoxin orally	23%	15%	23%	22%
Amiodarone to slow heart rate	75%	69%	75%	83%
Calcium channel blockers	7%	4%	9%	9%
AVN ablation	94%	78%	91%	67%
Treatment options for rhythm control				
AF ablation	86%	34%	79%	42%
Amiodarone	93%	96%	92%	85%
Flecainide	2%	0%	4%	7%
Sotalol	7%	2%	10%	11%
Repeated EC	29%	22%	22%	23%
Imaging and ablation				
Systematic atrial imaging to check the presence of thrombus	91%		90%	
In cases with typical atrial flutter, ablation is the initial approach	100%		100%	

The percentages indicate the proportion of the panel agreeing or not with each statement. Green cells show statements with positive convergence, while dark green cells highlight strong positive convergence (≥50% strongly agree). Red cells denote negative convergence, and dark red cells indicate strong negative convergence (≥50% strongly disagree). White cells represent statements that did not reach convergence after 2 rounds.

AF = atrial fibrillation; AL = amyloid light-chain; ATTRv = hereditary transthyretin amyloidosis; ATTRwt = wild-type transthyretin amyloidosis; AVN = atrioventricular node; EC = electrical cardioversion; NAC = National Amyloidosis Centre.

For patients experiencing AF recurrence after EC while on amiodarone, a rate control strategy was favored, irrespective of symptoms. The panel supported the use of amiodarone or atrioventricular node ablation for rate control, while discouraging oral digoxin and calcium channel blockers. Beta-blockers in progressive doses were also discouraged, particularly in advanced disease stages, due to a lack of consensus on their effectiveness.

### Prophylactic implantation of cardiac devices in asymptomatic CA patients

In this study, asymptomatic CA patients were those without symptoms such as syncope. For asymptomatic ATTR patients with conduction disorders, the panel favored proactive intervention and recommended management strategies over clinical monitoring except for patients with narrow QRS and first-degree atrioventricular block (AVB I) with a PR interval of 200 to 250 ms, where more intensive management was not deemed necessary (Table 2). The panel favored cardiac device implantation for patients with complete left bundle branch block and an AVB I with PR >250 ms, trifascicular block (AVB I + complete right bundle branch block [RBBB] + left anterior or posterior fascicular block), progressive conduction disorders, tachycardia-bradycardia syndrome, or sinus node dysfunction with diurnal pauses of 3 to 6 seconds. In contrast, PM implantation was not advised for patients with a narrow QRS complex, complete RBBB and AVB I with a PR interval between 200 and 250 ms, or nocturnal paroxysmal AV block II Mobitz 1.

**Table 2:** Electrophysiology Studies and Implantation of Devices in Patients With ATTRv, ATTRwt, or AL Cardiac Amyloidosis

	Management Strategies			
	PM/ICD	Electrophysiology Study	Long-Term Monitoring	Nothing
ATTRv or ATTRwt				
Narrow QRS and AV block I with PR between 200 and 250 ms	0%	27%	35%	67%
Narrow QRS and AV block I with PR >250 ms	21%	34%	49%	21%
QRS >120 ms, right bundle branch block, AV block I with PR between 200 and 250 ms	31%	71%	61%	13%
QRS >120 ms, left bundle branch block, AV block I with PR between 200 and 250 ms	49%	63%	61%	9%
QRS >120 ms, right bundle branch block, AV block I with PR >250 ms	65%	64%	47%	7%
QRS >120 ms, left bundle branch block, AV block I with PR >250 ms	75%	60%	43%	4%
Trifascicular block	80%	30%	24%	2%
Evolving conductive disorders	80%	32%	27%	2%
Paroxysmal diurnal transition to AV block II Mobitz 1	56%	50%	57%	5%
Paroxysmal nocturnal transition to AV block II Mobitz 1	32%	46%	47%	5%
Tachycardia-bradycardia syndrome	88%	11%	25%	5%
Sinus node dysfunction with diurnal pauses between 3 and 6 sec	84%	14%	33%	7%
If higher NAC stage, your management will be more intensive	71%			
AL				
Narrow QRS and AV block I with PR between 200 and 250 ms	6%	30%	39%	45%
Narrow QRS and AV block I with PR >250 ms	25%	31%	61%	17%
QRS >120 ms, right bundle branch block, AV block I with PR between 200 and 250 ms	32%	69%	59%	10%
QRS >120 ms, left bundle branch block, AV block I with PR between 200 and 250 ms	56%	59%	58%	10%
QRS >120 ms, right bundle branch block, AV block I with PR >250 ms	63%	63%	51%	8%
QRS >120 ms, left bundle branch block, AV block I with PR >250 ms	66%	55%	45%	4%
Trifascicular block	73%	36%	18%	4%
Evolving conductive disorders	83%	36%	23%	2%
Paroxysmal diurnal transition to AV block II Mobitz 1	53%	52%	41%	6%
Paroxysmal nocturnal transition to AV block II Mobitz 1	35%	38%	41%	6%
Tachycardia-bradycardia syndrome	83%	13%	30%	2%
Sinus node dysfunction with diurnal pauses between 3 and 6 sec	90%	15%	30%	2%
If higher European staging, your management will be more intensive	71%			

The percentages indicate the proportion of the panel agreeing or not with each statement. Green cells show statements with positive convergence, while dark green cells highlight strong positive convergence (≥50% strongly agree). Red cells denote negative convergence, and dark red cells indicate strong negative convergence (≥50% strongly disagree). White cells represent statements that did not reach convergence after 2 rounds.

AV block I = first-degree atrioventricular block; ICD = implantable cardioverter-defibrillator; Mobitz 1 = Mobitz type I second-degree atrioventricular block; ms = milliseconds; PM = pacemaker; sec = seconds; other abbreviations as in Table 1.

Electrophysiological studies were suggested only for patients with complete RBBB and AVB I with PR intervals between 200 and 250 ms.

The panel's voting highlighted a nuanced approach to cardiac device management. For most scenarios, there was limited agreement on the necessity of long-term monitoring. However, a negative consensus was reached in cases involving trifascicular block, progressive conduction disorders, tachycardia-bradycardia syndrome, or sinus node dysfunction with diurnal pauses of 3 to 6 seconds, situations where the implantation of a PM was strongly favored. The decision to intensify implantation management was guided by the disease stage as determined by the panel. The decision to implant a defibrillator was influenced by factors such as left ventricular ejection fraction (LVEF) and the presence of nonsustained ventricular tachycardia, regardless of pacing indication (Table 3). Global longitudinal strain did not influence these decisions. For ventricular pacing, the panel determined that the decision for CRT was influenced by LVEF adopting a cutoff of 50%, with the expected pacing percentage also affecting CRT eligibility.

**Table 3:** Factors Influencing Choices to Implant a Cardiac Device in Patients With ATTRv, ATTRwt, or AL Cardiac Amyloidosis

	ATTRv or ATTRwt	AL
Defibrillation		
Having an indication for pacing		
LVEF	80%	85%
GLS	21%	23%
NSVT	77%	81%
Not having an indication for pacing		
LVEF	76%	85%
GLS	30%	20%
NSVT	73%	75%
The hereditary nature of ATTR	20%	
Cardiac resynchronization therapy		
Having an indication for ventricular pacing		
LVEF	88%	92%
If so, a cutoff at 50% is appropriate	69%	70%
GLS	32%	32%
If so, a cutoff at 14% is appropriate	80%	75%
Expected pacing percentage influences a decision for CRT	87%	92%

The percentages indicate the proportion of the panel agreeing or not with each statement. Green cells show statements with positive convergence, while dark green cells highlight strong positive convergence (≥50% strongly agree). Red cells denote negative convergence, and dark red cells indicate strong negative convergence (≥50% strongly disagree). White cells represent statements that did not reach convergence after 2 rounds.

ATTR = transthyretin amyloidosis; CRT = cardiac resynchronization therapy; GLS = global longitudinal strain; LVEF = left ventricular ejection fraction; NSVT = nonsustained ventricular tachycardia; other abbreviations as in Table 1.

For AL patients, the results closely mirrored those observed in ATTR. However, the panel was unable to reach a consensus on PM implantation for patients presenting with a narrow QRS complex and AVB I with a PR interval exceeding 250 ms.

### Management strategies including anticoagulation therapy in CA patients in sinus rhythm

For AL patients in sinus rhythm, regular 24-hour Holter monitoring was advised by the panel in all scenarios. Long-term monitoring was suggested for patients with a history of stroke or transient ischemic attack (TIA) or frequent premature atrial contractions (PACs) between 1,000 and 10,000 per 24 hours. Similar results applied to ATTR patients, where monitoring was also suggested for PACs exceeding 1,000 per 24 hours (Table 4). However, long-term monitoring was not favored for AL patients with an exclusive E wave mitral profile, restrictive mitral profile, or CHA_2_DS_2_-VASc scores ≥3. The same was true for ATTR patients with similar conditions including a LVEF ≤50%.

**Table 4:** Management Strategies and Intensity in Patients With Cardiac Amyloidosis in Sinus Rhythm

	Management Strategies			Management Intensity	
	Regular 24 h Holter	Long-Term Monitoring	Anticoagulants	If Higher Stage	If Bursts of Arrhythmia
ATTRv or ATTRwt					
History of stroke with territory on brain imaging compatible with cardioembolic event	84%	82%	82%	80%	
History of recent TIA (<1 y)	77%	87%	75%	72%	
Atrial fibrillation detection on implantable device <6 min			80%	87%	
Mitral pattern with exclusive E wave	78%	26%	68%	68%	
Restrictive mitral inflow pattern	76%	20%	31%	60%	
CHA_2_DS_2_-VASc score ≥3, with no other thromboembolic risk factors (apart from amyloidosis)	79%	33%	20%	48%	
LVEF ≤50%	79%	28%	31%	31%	
LVEF ≤30%	79%	37%	55%	69%	
ECG Holter detection with PACs between 500 and 1,000 per 24 h	86%	55%	10%	69%	73%
ECG Holter detection with PACs between 1,000 and 10,000 per 24 h	91%	70%	51%	75%	84%
ECG Holter detection with PACs >10,000 PACs per 24 h	86%	73%	76%	83%	87%
AL					
History of stroke with territory on brain imaging compatible with cardioembolic event	75%	89%	83%	83%	
History of recent TIA (<1 y)	79%	94%	72%	80%	
Atrial fibrillation detection on implantable device <6 min			81%	78%	
Mitral pattern with exclusive E wave	75%	36%	65%	76%	
Restrictive mitral inflow pattern	81%	23%	25%	44%	
CHA_2_DS_2_-VASc score ≥3, with no other thromboembolic risk factors (apart from amyloidosis)	89%	32%	26%	40%	
LVEF ≤50%	85%	23%	28%	25%	
LVEF ≤30%	85%	47%	50%	65%	
ECG Holter detection with PACs between 500 and 1,000 per 24 h	91%	37%	24%	20%	70%
ECG Holter detection with PACs between 1,000 and 10,000 per 24 h	91%	70%	55%	71%	83%
ECG Holter detection with PACs >10,000 PACs per 24 h	89%	65%	76%	75%	87%

The percentages indicate the proportion of the panel agreeing or not with each statement. Green cells show statements with positive convergence, while dark green cells highlight strong positive convergence (≥50% strongly agree). Red cells denote negative convergence. White cells represent statements that did not reach convergence after 2 rounds.

CHA_2_DS_2_-VASc = a clinical prediction rule for estimating the risk of stroke in patients with atrial fibrillation; ECG = electrocardiogram; PACs = premature atrial contractions; TIA = transient ischemic attack; other abbreviations as in Tables 1 and 3.

Anticoagulation therapy was supported for AL and ATTR patients with a history of stroke or TIA, AF of <6 minutes detected via cardiac devices (implantable cardiac monitor or PM/ICD), an exclusive E wave mitral profile, or PACs exceeding 10,000 per 24 hours. For ATTR patients, anticoagulation was also considered appropriate when an exclusive E wave mitral profile was present. Conversely, anticoagulation therapy was not favored for AL or ATTR patients with a restrictive mitral profile, CHA_2_DS_2_-VASc scores ≥3, LVEF ≤50%, or PAC burdens between 500 and 1,000 per 24 hours.

More intensive management strategies, including frequent Holter monitoring or rapid anticoagulation initiation, were advised for ATTR patients with higher NAC staging scores, except for those with restrictive mitral profiles, CHA_2_DS_2_-VASc scores of 3 or higher, or LVEF ≤50%. Similar trends were observed in AL, where higher European staging scores prompted more intensive management strategies, except for patients with restrictive mitral profiles, CHA_2_DS_2_-VASc scores ≥3, or LVEF ≤50%, but also with PAC burdens between 500 and 1,000 per 24 hours.

## Discussion

In this study, we examined expert approaches to the management of AF and flutter, indications for cardiac device implantation, and anticoagulation therapy in sinus rhythm patients in patients with AL and ATTR including ATTRwt and ATTRv forms (Central Illustration). The importance of this Delphi study lies in its multidisciplinary approach, involving both cardiologists and electrophysiologists, to reflect current French practices in managing CA.

**Central Illustration:**
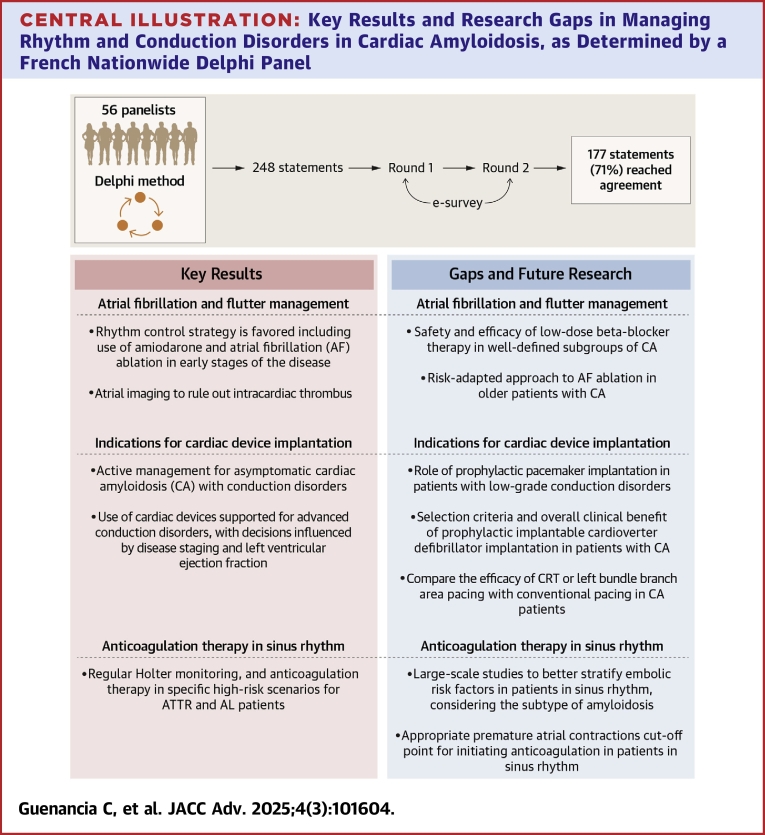
Key Results and Research Gaps in Managing Rhythm and Conduction Disorders in Cardiac Amyloidosis, as Determined by a French Nationwide Delphi Panel AL = amyloid light-chain; ATTR = transthyretin amyloidosis; CRT = cardiac resynchronization therapy.

### AF and flutter management

The prevalence of AF in CA is not yet fully established; however, a retrospective study on all types of CA demonstrated a prevalence of 44% compared to an estimated AF prevalence of 1% in the general population.^19^^,^^20^ Furthermore, this percentage increases with a higher NAC score in ATTR patients.^21^

Recent research provides further insight into the impact of AF on mortality rates among patients with CA. Notably, a retrospective study involving 382 ATTR patients demonstrated similar mortality rates in the presence or absence of AF,^19^ likely influenced by the high mortality of the pathology itself. Another study on AL and ATTR patients yielded similar results, corroborating these findings.^22^

When managing AF and flutter in CA, a rate control strategy focuses on controlling heart rate, while a rhythm control strategy aims to restore normal sinus rhythm.^23^ The panel recommended rhythm control for the first AF episode, however for recurrences, the focus shifts to rate control; although for AL amyloidosis, both strategies were considered for symptomatic recurrences.

It has been shown that first-line rhythm control may be appropriate for patients with recent-onset paroxysmal AF, particularly those experiencing adverse symptoms, and even asymptomatic patients as it may uncover previously unnoticed symptoms.^13^^,^^24^ Amiodarone was favorably endorsed as a therapeutic option within a rhythm control strategy by the panel, aligning with the 2021 European Society of Cardiology (ESC) guidelines for CA.^15^ AF ablation was also considered a suitable treatment option by our panel; although ablation is not favored for patients in the most advanced stages of the disease.^25^^,^^26^ Previous studies have demonstrated that optimal outcomes are achieved when the procedure is performed at an early stage of ATTR, as AF recurrence rates are 36% at NAC stage I or II compared to 90% at stage III.^22^ However, no published data exist regarding AF ablation in patients with AL amyloidosis. While the panel supports considering AF ablation in this population—similar to its use in ATTR amyloidosis—the timing of the intervention, which was not addressed by the panel, warrants careful deliberation. Chemotherapy should take precedence in these patients, given the urgency of addressing the underlying disease. During chemotherapy, rhythm control can be effectively managed with amiodarone. If AF persists or recurs following successful suppression of immunoglobulin light chains, catheter ablation may then be pursued as part of the rhythm control strategy. This sequential approach aligns with the need to balance disease-modifying treatment with symptom management. Regarding other rhythm control treatments, a negative consensus was reached for flecainide, sotalol, and repeated EC, as well documented by others.^13^^,^^27^^,^^28^

Repeated EC was not advised by our panel due to the lack of specific guidelines, urging a personalized approach, case-by-case approach. Misleading electrocardiogram (ECG) morphology complicates diagnosis, and careful consideration of the risk-benefit ratio, symptoms, and patient preferences are essential. Studies have shown that patients with CA face higher risks of postablation complications, net adverse clinical events, and mortality. It is important to note that the study's findings may be limited, as it did not distinguish between paroxysmal and persistent AF.

For rate control, the panel agrees on the use of both amiodarone to slow down the heart rate and AV node ablation, as described by others.^29^^,^^30^ The use of digoxin was not favored by our panel, as others demonstrated that it should be used cautiously due to its association with increased mortality.^15^^,^^31^^,^^32^ Historically, digoxin was considered contraindicated in CA due to in vitro evidence suggesting its binding to amyloid fibrils. However, more recent data indicate that digoxin can be cautiously used for rate control in AF in carefully monitored ATTR patients. The absence of large-scale, prospective studies on digoxin in CA, combined with long-standing concerns about its potential toxicity, likely contributed to the lack of consensus among the panel on this issue.^32^^,^^33^ Similarly, calcium channel blockers are generally poorly tolerated by CA patients, and experts on our panel reached a strong negative consensus on their use. These medications may interact with amyloid fibrils, leading to toxicity and potentially exacerbating heart failure.^31^^,^^34^ Current recommendations and position papers advise against the routine use of beta-blockers in initial treatment, though this remains debated within the medical community. At normal doses, beta-blockers can often lead to low cardiac output, fatigue, conduction disturbances, hypotension, and even syncope. Importantly, when beta-blockers are deemed necessary in early stages of the disease, it is critical to maintain low dosages to minimize potential adverse effects. However, results in different centers yield different results highlighting the need for further studies on low-dose usage.^35^^,^^36^ Beta-blocker therapy should be assessed on a case-by-case basis, tailored to the patient's individual response to treatment.

Thrombus management poses a significant challenge, necessitating systematic imaging to detect their presence. This is particularly critical for patients requiring ablation or cardioversion, as 14% to 40% of amyloidosis patients present with thrombi despite intensive anticoagulation.^28^^,^^37^ However, these studies were conducted on a small number of patients, and there is a need for confirmation through larger-scale cohorts.

### Indications for cardiac device implantation

The implantation of devices such as PM and ICD in the management of conduction and rhythm disorders in patients with CA is both complex and crucial.^38^ Standard follow-up protocols are insufficient for these patients due to the high prevalence of conduction disorders.^22^ Notably, studies have shown that approximately 10% to 15% of patients with CA (either AL or ATTR) develop high-risk conduction disorders requiring PM implantation.^39^^,^^40^ For example, in the study by Donnellan et al^41^ involving 369 ATTR patients, 9.5% had a PM before the diagnosis, and 11% had one implanted during follow-up (of 28 months). However, most studies on the progression of conduction disorders in patients with CA have been performed in cohorts where disease-modifying treatments were not available. Consequently, it remains unclear whether these treatments can reduce the overall risk of progression to advanced conduction disorders requiring intervention.

In our study, experts considered that management strategies varied based on NAC and European staging scores. While previous research highlighted increased mortality risk associated with NAC staging,^22^ similar research is lacking for European staging, signaling a need for further studies. For the highest-risk conduction disorders, the panel's agreement on the need of PM implantation is expected. However, management practices for moderate disorders varied, ranging from electrophysiological exploration to long-term monitoring and device implantation. The panel was not queried on treatment sequence or type of device for implantation, which warrants future exploration. However, for all patients except those with narrow QRS complexes and PR intervals <250 ms, there is a negative consensus on taking no action; as for the exception mentioned, close monitoring via Holter ECG deemed sufficient.^31^ The panel suggested the use of electrophysiologic study in the case of a complete RBBB + PR interval between 200 and 250 ms, although scientific evidence is lacking regarding the prognostic value of electrophysiological study measurements in predicting the development of high-grade AVB during follow-up.

The decision to implant an ICD was shown to be generally guided by the LVEF and the number of nonsustained ventricular tachycardia episodes in our study. This approach aligns with clinical observations despite the lack of specific ESC guidelines and limited literature.^37^ The ESC guidelines recommend that an ICD should be considered in patients with CA and hemodynamically not-tolerated ventricular tachycardia with a full understanding of the risks of nonarrhythmic deaths and noncardiac deaths.^37^ Kharoubi et al^42^ investigated mortality in AL and ATTR amyloidosis patients, revealing that 30% of patients died, with 65% of these deaths being of cardiovascular origin. The study highlighted that LVEF does not accurately stratify the risk of sudden death in CA patients. On the other hand, multiple retrospective studies have explored the use of a defibrillator in CA patients. Hamon et al^43^ found that 27% received appropriate therapy, yet an equivalent percentage died. This raises questions about the effectiveness of defibrillators in improving survival outcomes for CA patients.

For CRT, decisions are guided by the same criteria (LVEF, but not global longitudinal strain) and the expected ventricular pacing rate. Although international guidelines exist for other conditions, a few clinical studies have suggested benefits for patients with CA.^44^^,^^45^ Regarding the expected rate of ventricular pacing required to consider CRT, we did not request the panel to provide an exact figure, as this remains a subject of ongoing debate in the general population. However, it is generally recognized that a pacing threshold of 20% to 40% represents the minimum level of right ventricular stimulation capable of inducing pacing-induced cardiomyopathy.^46^ The nonresponder rate remains higher compared to patients without amyloidosis, necessitating ongoing evaluation of these practices. In the study by Rehorn et al^47^, 96 ± 1% of patients were PM-dependent at 5 years, with indications including sinus node dysfunction (27%), AVB (64%), and tachycardia-bradycardia syndrome (9%). This highlights the need to explore the potential benefits of early CRT before the onset of left ventricular dysfunction. Further research is needed to evaluate its clinical implications.

### Anticoagulation therapy in sinus rhythm patients

Multiple studies have demonstrated that thromboembolic events, including ischemic strokes, TIA, cerebrovascular events, and intracardiac thrombi, are prevalent in systemic amyloidosis, particularly in CA.^22^^,^^28^^,^^48^^,^^49^ Anticoagulation in the context of CA remains a particular challenge, especially for patients in sinus rhythm, as the literature lacks robust evidence firmly supporting this usage.^31^^,^^48^ Nonetheless, this practice is often justified based on clinical experience due to the high risk of cardioembolic events, even without evidence of arrhythmia in the presence of an atrial contractile dysfunction marker.^22^^,^^50^ Indication for oral anticoagulation in patients with CA and AF is normally advised regardless of ChA_2_DS_2_-VASc score.^49^^,^^51^ Capelli et al^48^ recently reported that 32% of patients with CA (AL, ATTRv, or ATTRwt) who suffered from arterial thromboembolic event, were in sinus rhythm without a history of AF. Their findings also suggested that patients in sinus rhythm with a CHA_2_DS_2_-VASc score of 3 or more might be a risk factor for arterial thromboembolic event, thereby warranting anticoagulation. However, in our study, expert agreement opposed the use of anticoagulation in sinus rhythm patients with a CHA_2_DS_2_-VASc score of 3 or more without additional thromboembolic risk factors beyond amyloidosis.

The panel of experts considered anticoagulation without AF reasonable in certain cases, such as recent stroke or TIA, AF detection <6 minutes, or exclusive E-wave mitral inflow, despite limited evidence. Future research should focus on identifying echocardiographic parameters of atrial contractile dysfunction that are associated with increased risk of stroke or TIA in sinus rhythm. Regarding prior stroke, although the sentence submitted to the panel specifically referred to recent stroke, we acknowledge that it does not fully reflect the intent of the panel's statement. The recommendation to consider anticoagulation should exclude strokes with a clear noncardioembolic etiology, such as those caused by atheromatous disease, small vessel disease, or arterial dissection. A more precise phrasing, such as “recent stroke (cardioembolic or cryptogenic) or TIA,” would have been more appropriate. Considering that the initial studies examining thromboembolic risk in CA identified AL amyloidosis as a predictive factor for the development of intracardiac thrombi, the inability to achieve an agreement within the panel on this subject is noteworthy.^52^^,^^53^ This may be explained by the relatively low prevalence of AL amyloidosis indicating that experts likely have more experience with treating ATTR patients.^51^ The persistent high risk of thromboembolic events in patients with CA underscores the importance of individualized discussions regarding the benefits of thrombosis prevention and the potential hemorrhagic risks associated with effective anticoagulation.^31^

The expert panel agrees against anticoagulation in cases of PACs burden <1,000 per 24 hours; but endorse close monitoring between 1,000 and 10,000. Anticoagulation is advised beyond 10,000 PACs per 24 hours. It is important to note that within the range of 1,000 to 10,000 PACs per 24 hours, additional measures should have been taken to establish a more precise threshold. Establishing a specific threshold for the number of PACs to predict AF would be highly advantageous in clinical practice.

Management becomes more intense in more advanced stages of the disease, with more frequent follow-up Holter monitoring and earlier initiation of anticoagulation therapy, consistent with literature that demonstrates a higher incidence of cardiovascular events as the disease progresses.^17^^,^^22^ Additionally, intense management is warranted in the presence of arrhythmic bursts, further emphasizing the need for vigilant monitoring and timely intervention in these high-risk patients.

This study did not investigate the sequence of management strategies, allowing participants to evaluate each approach independently. Some strategies may be justified but not implemented simultaneously. For patients already on anticoagulation therapy, conducting Holter monitoring or long-term recordings offers no additional benefit. Additionally, resource limitations in some centers may affect management decisions, depending on the availability of specific monitoring services. However, regular 24-hour Holter ECG monitoring every 12 months for at-risk patients in sinus rhythm is consistent with current recommendations.^15^

### Strengths and limitations

The Delphi method, developed in the 1960s for military use, is now widely used as a reliable decision-making tool that gathers expert opinions anonymously, often via online platforms. It is valued for its inclusiveness, rigor, and efficiency, fostering personal investment and involvement in the research.^54^ The committee chose this method for its proven reliability, and frequent use in health care.^55^

This study is one of the first to investigate current practices for CA through expert opinions, drawing on the specific clinical expertise of cardiologists and electrophysiologists specializing in CA. Its strength lies in the multidisciplinary collaboration between the 2, offering diverse perspectives and a balanced consensus to guide clinical practice. The agreement achieved by the experts is crucial in shaping and informing future clinical approaches, reflecting their collective knowledge (Table 5).

**Table 5:** Key Insights and Findings From the Management of Rhythm and Conduction Disorders in Cardiac Amyloidosis Delphi Study

The management of atrial fibrillation and flutter • A rhythm control strategy is favored for first AF episodes regardless of symptoms, with a preference for AF ablation in patients with lower NAC or European stages, and the use of amiodarone irrespective of disease stage. • Systematic atrial imaging to check for intracardiac thrombus is strongly advocated, and ablation is proposed as a first-line approach for typical atrial flutter. • For the rate control strategy, the proposed treatments include amiodarone (for slowing heart rate) or AV node ablation.
Prophylactic implantation of cardiac devices in asymptomatic CA patients • Active management is proposed rather than just clinical monitoring for asymptomatic CA patients with conduction disorders, except for those with a narrow QRS and first-degree atrioventricular block (PR interval 200-250 ms). • Cardiac device implantation is favored for cases with complete LBBB and AV block I with PR >250 ms for ATTR patients only. Additionally, it is favored for cases with trifascicular block, progressive conduction disorders, tachycardia-bradycardia syndrome, or sinus node dysfunction with diurnal pauses (3-6 s) for ATTR and AL patients. • Electrophysiological studies are favored only for complete RBBB or first-degree AV block (PR interval 200-250 ms) for ATTR and AL patients. • LVEF and NSVT are key criteria for defibrillator implantation. For CRT, LVEF cutoff is 50%, and the expected pacing percentage also influences the decision.
Anticoagulation therapy in CA patients in sinus rhythm • Regular 24-h Holter monitoring is necessary for ATTR and AL patients in sinus rhythm. • Long-term monitoring is preferred for those with a history of stroke/TIA or PACs between 1,000 and 10,000 per 24 h for AL patients. For ATTR patients, monitoring is advised for the same conditions including PACs >1,000 per 24 h. • Anticoagulants are advised for patients with a history of stroke/TIA, AF <6 min, or PACs >10,000 per 24 h for AL patients. For ATTR patients, anticoagulation is advised for the same conditions including an exclusive E wave mitral profile. • Anticoagulants are not favored for ATTR or AL patients with restrictive mitral patterns, CHA_2_DS_2_-VASc score ≥3, LVEF ≤50%, or PACs of 500-1,000 per 24 h. • For ATTR patients, more intensive management is warranted for higher NAC staging patients, except for those with restrictive mitral inflow pattern, CHA_2_DS_2_-VASc score ≥3, or LVEF ≤50%. • For AL patients, more intensive management is warranted for higher European staging patients, except for those with restrictive mitral inflow pattern, CHA_2_DS_2_-VASc score ≥3, or LVEF ≤50%, or PACs of 500-1,000 per 24 h.

AV = atrioventricular; CA = cardiac amyloidosis; LBBB = left bundle branch block; other abbreviations as in Tables 1, 3, and 4.

However, variations in clinical practices and the availability of procedures across different countries made an international Delphi study challenging. Despite the specialized knowledge of the participating French physicians, they identified several significant barriers to accurately managing rhythm and conduction disorders in CA. These challenges are probably encountered in other countries as well.

Our results highlighted current practices but emphasized the need to tailor treatments to individual patient profiles and age-specific factors. Experts emphasized the importance of a multidisciplinary approach to patient care, especially involving geriatric cardiologists for older patients, and advocated for shared medical decision-making due to the complexity and risks of interventions like AF ablation.^22^^,^^56^ This collaboration is essential to identify gaps especially in the absence of established guidelines (Table 6).

**Table 6:** Key Gaps and Future Research Identified in the Management of Rhythm and Conduction Disorders in Cardiac Amyloidosis Delphi Study

Atrial fibrillation and flutter management • Prospective studies should evaluate the safety and efficacy of low-dose beta-blocker therapy in well-defined subgroups of CA patients for controlling heart rate or rhythm. • Evaluating a risk-adapted approach to AF ablation in older patients with CA, incorporating a comprehensive geriatric assessment, is essential.
Indications for cardiac devices implantation • The role of prophylactic PM implantation in patients with low-grade conduction disorders remains to be demonstrated. This should be compared to alternative strategies, such as close monitoring with implantable loop recorders or electrophysiological studies. • The selection criteria and overall clinical benefit of prophylactic ICD implantation in patients with CA are yet to be clearly defined. • Prospective studies are necessary to compare the efficacy of CRT or left bundle branch area pacing with conventional pacing in CA patients, particularly those with a high expected ventricular pacing percentage or complete LBBB.
Anticoagulation therapy in sinus rhythm patients • Large-scale studies are needed to better stratify embolic risk factors in patients with sinus rhythm, considering the subtype of amyloidosis. • Research is required to determine the appropriate PAC cutoff point for initiating anticoagulation in patients with sinus rhythm.

Abbreviations as in Tables 1, 2, 3, 4, and 5.

Despite the pathophysiological differences between AL and ATTR, our study found surprisingly similar management approaches for both conditions. This is notable given the distinct mechanisms underlying each disease.^10^ Although response bias cannot be entirely ruled out, the low prevalence of AL suggests that experts are likely to have more experience managing ATTR.

## Conclusions

The existing literature on the management of rhythm and conduction disorders in CA is limited, primarily comprising small case series from single centers. This modified Delphi study aimed to establish expert agreement on current practices in France. Approximately 70% of the proposed statements achieved agreement, reflecting strong alignment on anticoagulation therapy, AF management, and implantable cardiac devices. However, the study also identified gaps in the understanding and management of rhythm and conduction disorders in CA. Future research should address these gaps to develop comprehensive, evidence-based guidelines and recommendations for effective electrophysiological CA management.

Perspectives **COMPETENCY IN MEDICAL KNOWLEDGE:** In CA patients, regular Holter monitoring and anticoagulation are prescribed in high-risk cases. Rhythm control strategies, such as amiodarone and AF ablation, are performed in early disease stages. Cardiac devices are used for advanced conduction disorders, with decisions based on disease stage and LVEF.

**TRANSLATIONAL OUTLOOK:** Further research is needed to address the gaps identified in the management of rhythm and conduction disorders in CA and to develop comprehensive, evidence-based guidelines for anticoagulation therapy, AF management, and the use of implantable cardiac devices.

## Funding support and author disclosures

This Delphi study was sponsored by Pfizer, France. Prof Guenancia has received personal fees from MicroPort CRM, Medtronic, AstraZeneca, Bristol Myers Squibb (BMS), Pfizer, Abbott, and AOP Pharma. Dr Lequeux reports financial ties with Pfizer. Dr Amara has received consulting and speaker fees from Pfizer, Biotronik, Medtronic, Boston Scientific, Microport, and Abbott. Dr Buiciuc is associated with Pfizer in relation to this study. Prof Damy reports relationships with Alnylam, Alexion, AstraZeneca, Bayer, Pfizer, BridgeBio, Neurimmune, Prothena, and Novo Nordisk. Dr Eicher has received consulting fees and honoraria from Pfizer and Alnylam. Prof Lairez has served as a consultant and speaker for Alnylam, Amicus, and Pfizer, a consultant for AstraZeneca, and as a speaker for BMS and Siemens Healthineers. Prof Lellouche has received speaker fees from BMS and Pfizer, and consulting fees from Medtronic, Abbott, and Boston Scientific. Dr Oghina has received honoraria from Pfizer, Bayer, Alnylam, and AstraZeneca. All other authors have reported that they have no relationships relevant to the contents of this paper to disclose.
