# Supplementary material for: Management of Rhythm and Conduction Disorders in Cardiac Amyloidosis: A French Nationwide Delphi Study
Source: JACC Adv. 2025 Feb 15;4(3):101604. doi: 10.1016/j.jacadv.2025.101604 (PMC11872529; doi:10.1016/j.jacadv.2025.101604)
Supplement: Supplemental Table 1 [file mmc1.docx]

**Supplemental Table 1.** Comprehensive list of the panel respondents from the “Rhythm Disorders in Cardiac Amyloidosis Expert Group,” categorized by French Regions.

| **Title** | **Family name** | **Name** | **Region in France** |
| --- | --- | --- | --- |
| Dr | Bouchot | Océane | Auvergne-Rhône-Alpes |
| Dr | Gardey | Kevin | Auvergne-Rhône-Alpes |
| Dr | Guijarro | Damien | Auvergne-Rhône-Alpes |
| Dr | Hugon-Vallet | Elisabeth | Auvergne-Rhône-Alpes |
| Dr | Didier | Romain | Bourgogne-Franche-Comté |
| Dr | Eicher | Jean-Christophe | Bourgogne-Franche-Comté |
| Dr | Mouhat | Basile | Bourgogne-Franche-Comté |
| Pr | Donal | Erwan | Bretagne |
| Dr | Galand | Vincent | Bretagne |
| Dr | Socié | Pierre | Centre-Val de Loire |
| Dr | Inamo | Jocelyn | Martinique |
| Dr | Mussigbrodt | Andreas | Martinique |
| Dr | Costa | Jérôme | Grand Est |
| Dr | Dagrenat-Favreau | Charlotte | Grand Est |
| Pr | Huttin | Olivier | Grand Est |
| Dr | Kenizou | David | Grand Est |
| Dr | Von Hunolstein | Jean-Jacques | Grand Est |
| Pr | Lamblin | Nicolas | Hauts-de-France |
| Dr | Buiciuc | Otilia | Hauts-de-France |
| Dr | Menet | Aymeric | Hauts-de-France |
| Dr | Mouquet | Frédéric | Hauts-de-France |
| Dr | Traullé | Sarah | Hauts-de-France |
| Dr | Vermes | Emmanuelle | Hauts-de-France |
| Dr | Algalarrondo | Vincent | Île-de-France |
| Dr | Amara | Walid | Île-de-France |
| Dr | Baudinaud | Pierre | Île-de-France |
| Dr | Bodez | Diane | Île-de-France |
| Dr | Chadha | Gagan | Île-de-France |
| Dr | Coquard | Charlène | Île-de-France |
| Pr | Damy | Thibaud | Île-de-France |
| Dr | Dinanian | Sylvie | Île-de-France |
| Dr | Galat | Arnault | Île-de-France |
| Dr | Guendouz | Soulef | Île-de-France |
| Pr | Hagege | Albert | Île-de-France |
| Pr | Lellouche | Nicolas | Île-de-France |
| Dr | Moulin | Thibaut | Île-de-France |
| Dr | Surget | Elodie | Île-de-France |
| Pr | Wahbi | Karim | Île-de-France |
| Pr | Bauer | Fabrice | Normandie |
| Dr | Legallois | Damien | Normandie |
| Dr | Tournevache | Fabian | Normandie |
| Pr | Bouleti | Claire | Nouvelle-Aquitaine |
| Pr | Reant | Patricia | Nouvelle-Aquitaine |
| Pr | Garcia | Rodrigue | Nouvelle-Aquitaine |
| Dr | Gellen | Barnabas | Nouvelle-Aquitaine |
| Dr | Mathelie | Margaux | Nouvelle-Aquitaine |
| Dr | Cariou | Eve | Occitanie |
| Dr | Duparc | Alexandre | Occitanie |
| Dr | Eyharts | Damien | Occitanie |
| Pr | Lairez | Olivier | Occitanie |
| Pr | Roubille | François | Occitanie |
| Dr | Gueffet | Jean Pierre | Pays de la Loire |
| Dr | Jeanneteau | Julien | Pays de la Loire |
| Pr | Trochu | Jean-Noël | Pays de la Loire |
| Pr | Habib | Gilbert | Provence-Alpes-Côte d'Azur |
| Dr | Tartière | Jean-Michel | Provence-Alpes-Côte d'Azur |

**Supplemental Table 2.** Clinical experience of the panel of respondents

| **Do you manage patients with ATTRwt or ATTRv ?** | |
| --- | --- |
| Yes | 100% |
| No | 0% |
| **How many patients on average ?** | |
| Between 0 and 10 | 11% |
| Between 10 and 50 | 32% |
| More than 50 | 57% |
| **Do you manage patients with AL ?** | |
| Yes | 86% |
| No | 14% |
| **How many patients on average ?** | |
| Between 0 and 10 | 56% |
| Between 10 and 50 | 29% |
| More than 50 | 17% |
| **How many years of experience in managing these patients?** | |
| Less than 5 years | 29% |
| Between 5 and 10 years | 38% |
| Between 10 and 15 years | 23% |
| More than 15 years | 11% |

**Supplemental Document 1. Delphi questionnaire (English translation)**

**INTRODUCTION**

Welcome!

Today, we are presenting a new questionnaire titled DELPHI Management of Cardiac Rhythm Disorders.

This questionnaire is available on our website as part of a study on the management of cardiac rhythm disorders in patients with cardiac amyloidosis. Its objectives are to better understand your habits, practices, and opinions on this subject.

Your responses will be used exclusively for statistical purposes.

We estimate that completing the questionnaire will take approximately 30 minutes. We recommend completing the questionnaire in one sitting. Your responses will be saved temporarily, provided you use the same computer and web browser.

Thank you in advance for your participation.

- **Please indicate the code assigned to you:**

Code:

- **Do you agree to waive confidentiality regarding adverse events and share your contact information with our client for the purpose of reporting adverse events?** *(Select one)*

Yes

No

- **Are you?** *(Select one)*

Cardiologist

Electrophysiologist

- **In which department are you located?**

Department:

- **Do you manage patients with ATTRwt or ATTRv CM?** *(Select one)*

Yes

No

- **If yes, on average, how many patients do you manage?** *(Select one)*

Between 0 and 10

Between 10 and 50

More than 50

- **Do you manage patients with AL amyloidosis?** *(Select one)*

Yes

No

- **If yes, on average, how many patients do you manage?** *(Select one)*

Between 0 and 10

Between 10 and 50

More than 50

- **How many years of experience do you have managing these patients?** *(Select one)*

Less than 5 years

Between 5 and 10 years

Between 10 and 15 years

More than 15 years

**ANTICOAGULATION (ATTR)**

- **Indicate your level of agreement with the proposed management strategies for a patient with ATTRwt or ATTRv CM in sinus rhythm for each of the following scenarios, and indicate in the last two columns whether your management would be more aggressive in case of a higher NAC stage or the presence of bursts of arrhythmia:** *(The different strategies are not mutually exclusive; you provide your opinion on each strategy independently).

  Management Strategies*

|  | **Regular 24h Holter** | **Long term monitoring** | **Anti-coagulants** | **If higher NAC stage** | **If bursts of arrhythmia** |
| --- | --- | --- | --- | --- | --- |
| History of stroke with territory on brain imaging compatible with cardioembolic event | 1 – Strongly disagree  2 – Disagree  3 – Agree  4 – Strongly agree | 1 – Strongly disagree  2 – Disagree  3 – Agree  4 – Strongly agree | 1 – Strongly disagree  2 – Disagree  3 – Agree  4 – Strongly agree | 1 – Strongly disagree  2 – Disagree  3 – Agree  4 – Strongly agree |  |
| History of recent TIA (less than 1 year) | 1 – Strongly disagree  2 – Disagree  3 – Agree  4 – Strongly agree | 1 – Strongly disagree  2 – Disagree  3 – Agree  4 – Strongly agree | 1 – Strongly disagree  2 – Disagree  3 – Agree  4 – Strongly agree | 1 – Strongly disagree  2 – Disagree  3 – Agree  4 – Strongly agree |  |
| Atrial fibrillation detection on implantable device < 6 min |  |  | 1 – Strongly disagree  2 – Disagree  3 – Agree  4 – Strongly agree | 1 – Strongly disagree  2 – Disagree  3 – Agree  4 – Strongly agree |  |
| Mitral pattern with exclusive E wave | 1 – Strongly disagree  2 – Disagree  3 – Agree  4 – Strongly agree | 1 – Strongly disagree  2 – Disagree  3 – Agree  4 – Strongly agree | 1 – Strongly disagree  2 – Disagree  3 – Agree  4 – Strongly agree | 1 – Strongly disagree  2 – Disagree  3 – Agree  4 – Strongly agree |  |
| Restrictive mitral inflow pattern | 1 – Strongly disagree  2 – Disagree  3 – Agree  4 – Strongly agree | 1 – Strongly disagree  2 – Disagree  3 – Agree  4 – Strongly agree | 1 – Strongly disagree  2 – Disagree  3 – Agree  4 – Strongly agree | 1 – Strongly disagree  2 – Disagree  3 – Agree  4 – Strongly agree |  |
| CHA_2_DS_2_-VASc score ≥ 3, with no other thromboembolic risk factors (apart from amyloidosis) | 1 – Strongly disagree  2 – Disagree  3 – Agree  4 – Strongly agree | 1 – Strongly disagree  2 – Disagree  3 – Agree  4 – Strongly agree | 1 – Strongly disagree  2 – Disagree  3 – Agree  4 – Strongly agree | 1 – Strongly disagree  2 – Disagree  3 – Agree  4 – Strongly agree |  |
| LVEF ≤ 50% | 1 – Strongly disagree  2 – Disagree  3 – Agree  4 – Strongly agree | 1 – Strongly disagree  2 – Disagree  3 – Agree  4 – Strongly agree | 1 – Strongly disagree  2 – Disagree  3 – Agree  4 – Strongly agree | 1 – Strongly disagree  2 – Disagree  3 – Agree  4 – Strongly agree |  |
| LVEF ≤ 30% | 1 – Strongly disagree  2 – Disagree  3 – Agree  4 – Strongly agree | 1 – Strongly disagree  2 – Disagree  3 – Agree  4 – Strongly agree | 1 – Strongly disagree  2 – Disagree  3 – Agree  4 – Strongly agree | 1 – Strongly disagree  2 – Disagree  3 – Agree  4 – Strongly agree |  |
| ECG Holter detection with PACs between 500 and 1000 per 24h | 1 – Strongly disagree  2 – Disagree  3 – Agree  4 – Strongly agree | 1 – Strongly disagree  2 – Disagree  3 – Agree  4 – Strongly agree | 1 – Strongly disagree  2 – Disagree  3 – Agree  4 – Strongly agree | 1 – Strongly disagree  2 – Disagree  3 – Agree  4 – Strongly agree | 1 – Strongly disagree  2 – Disagree  3 – Agree  4 – Strongly agree |
| ECG Holter detection with PACs between 1000 and 10000 per 24h | 1 – Strongly disagree  2 – Disagree  3 – Agree  4 – Strongly agree | 1 – Strongly disagree  2 – Disagree  3 – Agree  4 – Strongly agree | 1 – Strongly disagree  2 – Disagree  3 – Agree  4 – Strongly agree | 1 – Strongly disagree  2 – Disagree  3 – Agree  4 – Strongly agree | 1 – Strongly disagree  2 – Disagree  3 – Agree  4 – Strongly agree |
| ECG Holter detection with PACs greater than 10000 PACs per 24h | 1 – Strongly disagree  2 – Disagree  3 – Agree  4 – Strongly agree | 1 – Strongly disagree  2 – Disagree  3 – Agree  4 – Strongly agree | 1 – Strongly disagree  2 – Disagree  3 – Agree  4 – Strongly agree | 1 – Strongly disagree  2 – Disagree  3 – Agree  4 – Strongly agree | 1 – Strongly disagree  2 – Disagree  3 – Agree  4 – Strongly agree |

*Definition of NAC*

*The "NAC" score (National Amyloidosis Center), developed by Gillmore et al., stratifies patients with ATTR amyloidosis into three survival groups based on NT-proBNP elevation >3000 ng/L and a reduction in estimated glomerular filtration rate (eGFR) calculated using the MDRD formula to <45 mL/min/1.73m².*


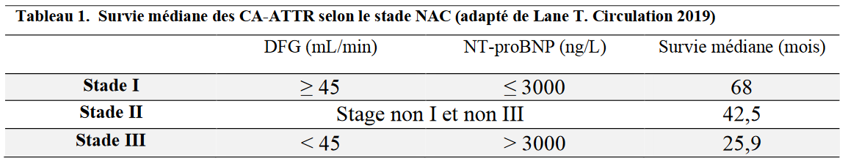


**AF AND FLUTTER (ATTR)**

- **Indicate your level of agreement with the proposed management strategies for atrial fibrillation in a patient with ATTRwt or ATTRv for each of the following scenarios:**

|  | **Rhythm control** | **Rate control** |
| --- | --- | --- |
| First episode of symptomatic AF | 1 – Strongly disagree  2 – Disagree  3 – Agree  4 – Strongly agree | 1 – Strongly disagree  2 – Disagree  3 – Agree  4 – Strongly agree |
| First episode of asymptomatic AF | 1 – Strongly disagree  2 – Disagree  3 – Agree  4 – Strongly agree | 1 – Strongly disagree  2 – Disagree  3 – Agree  4 – Strongly agree |
| After recurrence of symptomatic EC, on amiodarone | 1 – Strongly disagree  2 – Disagree  3 – Agree  4 – Strongly agree | 1 – Strongly disagree  2 – Disagree  3 – Agree  4 – Strongly agree |
| After recurrence of asymptomatic EC, on amiodarone | 1 – Strongly disagree  2 – Disagree  3 – Agree  4 – Strongly agree | 1 – Strongly disagree  2 – Disagree  3 – Agree  4 – Strongly agree |

- **Indicate your level of agreement:**

**If the NAC stage is higher, your management will be more aggressive.**
1 – Strongly disagree 2 – Disagree 3 – Agree 4 – Strongly agree

- **You have chosen a rate control strategy. For a patient with ATTRwt or ATTRv CM presenting with rapid AF and preserved LVEF, indicate your level of agreement with each proposed therapeutic option, and specify whether your management would be more aggressive if the NAC stage is higher**

|  |  | **If higher NAC stage** |
| --- | --- | --- |
| Beta-blockers in progressive doses | 1 – Strongly disagree  2 – Disagree  3 – Agree  4 – Strongly agree | 1 – Strongly disagree  2 – Disagree  3 – Agree  4 – Strongly agree |
| Digoxin orally | 1 – Strongly disagree  2 – Disagree  3 – Agree  4 – Strongly agree | 1 – Strongly disagree  2 – Disagree  3 – Agree  4 – Strongly agree |
| Amiodarone to slow heart rate | 1 – Strongly disagree  2 – Disagree  3 – Agree  4 – Strongly agree | 1 – Strongly disagree  2 – Disagree  3 – Agree  4 – Strongly agree |
| Calcium channel blockers | 1 – Strongly disagree  2 – Disagree  3 – Agree  4 – Strongly agree | 1 – Strongly disagree  2 – Disagree  3 – Agree  4 – Strongly agree |
| AVN ablation | 1 – Strongly disagree  2 – Disagree  3 – Agree  4 – Strongly agree | 1 – Strongly disagree  2 – Disagree  3 – Agree  4 – Strongly agree |

- **You have chosen a rhythm control strategy. For a patient with ATTRwt or ATTRv CM presenting with AF and preserved LVEF, indicate your level of agreement with each proposed therapeutic option, and specify whether your management would be more aggressive if the NAC stage is higher:**

|  |  | **If higher NAC stage** |
| --- | --- | --- |
| AF ablation | 1 – Strongly disagree  2 – Disagree  3 – Agree  4 – Strongly agree | 1 – Strongly disagree  2 – Disagree  3 – Agree  4 – Strongly agree |
| Amiodarone | 1 – Strongly disagree  2 – Disagree  3 – Agree  4 – Strongly agree | 1 – Strongly disagree  2 – Disagree  3 – Agree  4 – Strongly agree |
| Flecainide | 1 – Strongly disagree  2 – Disagree  3 – Agree  4 – Strongly agree | 1 – Strongly disagree  2 – Disagree  3 – Agree  4 – Strongly agree |
| Sotalol | 1 – Strongly disagree  2 – Disagree  3 – Agree  4 – Strongly agree | 1 – Strongly disagree  2 – Disagree  3 – Agree  4 – Strongly agree |
| Repeated EC | 1 – Strongly disagree  2 – Disagree  3 – Agree  4 – Strongly agree | 1 – Strongly disagree  2 – Disagree  3 – Agree  4 – Strongly agree |

- **You have chosen a cardioversion strategy for a patient with ATTRwt or ATTRv CM, who is effectively anticoagulated. Indicate your level of agreement with performing systematic atrial imaging to check presence of thrombus:**

1 – Strongly disagree 2 – Disagree 3 – Agree 4 – Strongly agree

- **For a patient with ATTRwt or ATTRv CM presenting with typical atrial flutter, indicate your level of agreement with performing first-line ablation:**

1 – Strongly disagree 2 – Disagree 3 – Agree 4 – Strongly agree

**IMPLANTATION (ATTR)**

- **Indicate your level of agreement with the proposed management approaches for an asymptomatic patient with ATTRwt or ATTRv CM in each of the following scenarios:** *(The different strategies are not mutually exclusive; you are providing your opinion on each strategy independently.)*

|  | **PM/ICD** | **Electrophysiology study** | **Long term monitoring** | **Nothing** |
| --- | --- | --- | --- | --- |
| Narrow QRS and AV block I with PR between 200 and 250 ms | 1 – Strongly disagree  2 – Disagree  3 – Agree  4 – Strongly agree | 1 – Strongly disagree  2 – Disagree  3 – Agree  4 – Strongly agree | 1 – Strongly disagree  2 – Disagree  3 – Agree  4 – Strongly agree | 1 – Strongly disagree  2 – Disagree  3 – Agree  4 – Strongly agree |
| Narrow QRS and AV block I with PR > 250 ms | 1 – Strongly disagree  2 – Disagree  3 – Agree  4 – Strongly agree | 1 – Strongly disagree  2 – Disagree  3 – Agree  4 – Strongly agree | 1 – Strongly disagree  2 – Disagree  3 – Agree  4 – Strongly agree | 1 – Strongly disagree  2 – Disagree  3 – Agree  4 – Strongly agree |
| QRS > 120 ms, right bundle branch block, AV block I with PR between 200 et 250 ms | 1 – Strongly disagree  2 – Disagree  3 – Agree  4 – Strongly agree | 1 – Strongly disagree  2 – Disagree  3 – Agree  4 – Strongly agree | 1 – Strongly disagree  2 – Disagree  3 – Agree  4 – Strongly agree | 1 – Strongly disagree  2 – Disagree  3 – Agree  4 – Strongly agree |
| QRS > 120 ms, left bundle branch block, AV block I with PR between 200 et 250 ms | 1 – Strongly disagree  2 – Disagree  3 – Agree  4 – Strongly agree | 1 – Strongly disagree  2 – Disagree  3 – Agree  4 – Strongly agree | 1 – Strongly disagree  2 – Disagree  3 – Agree  4 – Strongly agree | 1 – Strongly disagree  2 – Disagree  3 – Agree  4 – Strongly agree |
| QRS > 120 ms, right bundle branch block, AV block I with PR > 250 ms | 1 – Strongly disagree  2 – Disagree  3 – Agree  4 – Strongly agree | 1 – Strongly disagree  2 – Disagree  3 – Agree  4 – Strongly agree | 1 – Strongly disagree  2 – Disagree  3 – Agree  4 – Strongly agree | 1 – Strongly disagree  2 – Disagree  3 – Agree  4 – Strongly agree |
| QRS > 120 ms, left bundle branch block, AV block I with PR > 250 ms | 1 – Strongly disagree  2 – Disagree  3 – Agree  4 – Strongly agree | 1 – Strongly disagree  2 – Disagree  3 – Agree  4 – Strongly agree | 1 – Strongly disagree  2 – Disagree  3 – Agree  4 – Strongly agree | 1 – Strongly disagree  2 – Disagree  3 – Agree  4 – Strongly agree |
| Trifascicular block | 1 – Strongly disagree  2 – Disagree  3 – Agree  4 – Strongly agree | 1 – Strongly disagree  2 – Disagree  3 – Agree  4 – Strongly agree | 1 – Strongly disagree  2 – Disagree  3 – Agree  4 – Strongly agree | 1 – Strongly disagree  2 – Disagree  3 – Agree  4 – Strongly agree |
| Evolving conductive disorders | 1 – Strongly disagree  2 – Disagree  3 – Agree  4 – Strongly agree | 1 – Strongly disagree  2 – Disagree  3 – Agree  4 – Strongly agree | 1 – Strongly disagree  2 – Disagree  3 – Agree  4 – Strongly agree | 1 – Strongly disagree  2 – Disagree  3 – Agree  4 – Strongly agree |
| Paroxysmal diurnal transition to AV block II Mobitz 1 | 1 – Strongly disagree  2 – Disagree  3 – Agree  4 – Strongly agree | 1 – Strongly disagree  2 – Disagree  3 – Agree  4 – Strongly agree | 1 – Strongly disagree  2 – Disagree  3 – Agree  4 – Strongly agree | 1 – Strongly disagree  2 – Disagree  3 – Agree  4 – Strongly agree |
| Paroxysmal nocturnal transition to AV block II Mobitz 1 | 1 – Strongly disagree  2 – Disagree  3 – Agree  4 – Strongly agree | 1 – Strongly disagree  2 – Disagree  3 – Agree  4 – Strongly agree | 1 – Strongly disagree  2 – Disagree  3 – Agree  4 – Strongly agree | 1 – Strongly disagree  2 – Disagree  3 – Agree  4 – Strongly agree |
| Tachycardia-bradycardia syndrome | 1 – Strongly disagree  2 – Disagree  3 – Agree  4 – Strongly agree | 1 – Strongly disagree  2 – Disagree  3 – Agree  4 – Strongly agree | 1 – Strongly disagree  2 – Disagree  3 – Agree  4 – Strongly agree | 1 – Strongly disagree  2 – Disagree  3 – Agree  4 – Strongly agree |
| Sinus node dysfunction with diurnal pauses pause between 3 and 6 sec | 1 – Strongly disagree  2 – Disagree  3 – Agree  4 – Strongly agree | 1 – Strongly disagree  2 – Disagree  3 – Agree  4 – Strongly agree | 1 – Strongly disagree  2 – Disagree  3 – Agree  4 – Strongly agree | 1 – Strongly disagree  2 – Disagree  3 – Agree  4 – Strongly agree |

*Definitions*

***Trifascicular block****: A trifascicular block is an electrical conduction problem in the heart, specifically involving all three fascicles of the bundle branches that transmit electrical signals from the atrioventricular node to the ventricles.*

***Tachycardia-bradycardia syndrome*** *is a heart rhythm disorder that affects the atria of the heart. It is characterized by alterations in atrial tissue, which can lead to irregular heart rhythms of varying frequency. Symptoms may include palpitations, dizziness, fatigue, chest pain, and heart failure.*

- **Indicate your level of agreement for each criterion. For a patient with ATTRwt or ATTRv CM who has an indication for pacing, the following criteria influence your decision to implant a defibrillator**

**LVEF**
1 – Strongly disagree 2 – Disagree 3 – Agree 4 – Strongly agree

**GLS**
1 – Strongly disagree 2 – Disagree 3 – Agree 4 – Strongly agree

**NSVT**
1 – Strongly disagree 2 – Disagree 3 – Agree 4 – Strongly agree

- **Indicate your level of agreement for each criterion. For a patient with ATTRwt or ATTRv CM who does not have an indication for pacing, the following criteria influence your decision to implant a defibrillator:**

**LVEF**
1 – Strongly disagree 2 – Disagree 3 – Agree 4 – Strongly agree

**GLS**
1 – Strongly disagree 2 – Disagree 3 – Agree 4 – Strongly agree

**NSVT**
1 – Strongly disagree 2 – Disagree 3 – Agree 4 – Strongly agree

**The hereditary nature of ATTR**
1 – Strongly disagree 2 – Disagree 3 – Agree 4 – Strongly agree

- **Indicate your level of agreement. For a patient with ATTRwt or ATTRv CM who has an indication for ventricular pacing.**

**The LVEF will influence a decision for CRT:**

1 – Strongly disagree 2 – Disagree 3 – Agree 4 – Strongly agree

**A cut-off at 50% seems appropriate:**

1 – Strongly disagree 2 – Disagree 3 – Agree 4 – Strongly agree

**The Global Longitudinal Strain will influence a decision for CRT:**
1 – Strongly disagree 2 – Disagree 3 – Agree 4 – Strongly agree

**A cut-off at 14% seems appropriate:**

1 – Strongly disagree 2 – Disagree 3 – Agree 4 – Strongly agree

**The expected percentage of pacing will influence a decision for CRT:**
1 – Strongly disagree 2 – Disagree 3 – Agree 4 – Strongly agree

**ANTICOAGULATION (AL)**

- **Indicate your level of agreement with the proposed management strategies for a patient with AL amyloidosis in sinus rhythm for each of the following scenarios. Additionally, indicate in the last two columns whether your management approach would be more aggressive in the case of a higher European Staging score or the presence of runs of arrhythmias:** *(The different management strategies are not mutually exclusive; you are providing your opinion on each strategy independently.)

  Management strategies*

|  | **Regular 24h Holter** | **Long term monitoring** | **Anti-coagulants** | **If higher European stage** | **If bursts of arrhythmia** |
| --- | --- | --- | --- | --- | --- |
| History of stroke with territory on brain imaging compatible with cardioembolic event | 1 – Strongly disagree  2 – Disagree  3 – Agree  4 – Strongly agree | 1 – Strongly disagree  2 – Disagree  3 – Agree  4 – Strongly agree | 1 – Strongly disagree  2 – Disagree  3 – Agree  4 – Strongly agree | 1 – Strongly disagree  2 – Disagree  3 – Agree  4 – Strongly agree |  |
| History of recent TIA (less than 1 year) | 1 – Strongly disagree  2 – Disagree  3 – Agree  4 – Strongly agree | 1 – Strongly disagree  2 – Disagree  3 – Agree  4 – Strongly agree | 1 – Strongly disagree  2 – Disagree  3 – Agree  4 – Strongly agree | 1 – Strongly disagree  2 – Disagree  3 – Agree  4 – Strongly agree |  |
| Atrial fibrillation detection on implantable device < 6 min |  |  | 1 – Strongly disagree  2 – Disagree  3 – Agree  4 – Strongly agree | 1 – Strongly disagree  2 – Disagree  3 – Agree  4 – Strongly agree |  |
| Mitral pattern with exclusive E wave | 1 – Strongly disagree  2 – Disagree  3 – Agree  4 – Strongly agree | 1 – Strongly disagree  2 – Disagree  3 – Agree  4 – Strongly agree | 1 – Strongly disagree  2 – Disagree  3 – Agree  4 – Strongly agree | 1 – Strongly disagree  2 – Disagree  3 – Agree  4 – Strongly agree |  |
| Restrictive mitral inflow pattern | 1 – Strongly disagree  2 – Disagree  3 – Agree  4 – Strongly agree | 1 – Strongly disagree  2 – Disagree  3 – Agree  4 – Strongly agree | 1 – Strongly disagree  2 – Disagree  3 – Agree  4 – Strongly agree | 1 – Strongly disagree  2 – Disagree  3 – Agree  4 – Strongly agree |  |
| CHA_2_DS_2_-VASc score ≥ 3, with no other thromboembolic risk factors (apart from amyloidosis) | 1 – Strongly disagree  2 – Disagree  3 – Agree  4 – Strongly agree | 1 – Strongly disagree  2 – Disagree  3 – Agree  4 – Strongly agree | 1 – Strongly disagree  2 – Disagree  3 – Agree  4 – Strongly agree | 1 – Strongly disagree  2 – Disagree  3 – Agree  4 – Strongly agree |  |
| LVEF ≤ 50% | 1 – Strongly disagree  2 – Disagree  3 – Agree  4 – Strongly agree | 1 – Strongly disagree  2 – Disagree  3 – Agree  4 – Strongly agree | 1 – Strongly disagree  2 – Disagree  3 – Agree  4 – Strongly agree | 1 – Strongly disagree  2 – Disagree  3 – Agree  4 – Strongly agree |  |
| LVEF ≤ 30% | 1 – Strongly disagree  2 – Disagree  3 – Agree  4 – Strongly agree | 1 – Strongly disagree  2 – Disagree  3 – Agree  4 – Strongly agree | 1 – Strongly disagree  2 – Disagree  3 – Agree  4 – Strongly agree | 1 – Strongly disagree  2 – Disagree  3 – Agree  4 – Strongly agree |  |
| ECG Holter detection with PACs between 500 and 1000 per 24h | 1 – Strongly disagree  2 – Disagree  3 – Agree  4 – Strongly agree | 1 – Strongly disagree  2 – Disagree  3 – Agree  4 – Strongly agree | 1 – Strongly disagree  2 – Disagree  3 – Agree  4 – Strongly agree | 1 – Strongly disagree  2 – Disagree  3 – Agree  4 – Strongly agree | 1 – Strongly disagree  2 – Disagree  3 – Agree  4 – Strongly agree |
| ECG Holter detection with PACs between 1000 and 10000 per 24h | 1 – Strongly disagree  2 – Disagree  3 – Agree  4 – Strongly agree | 1 – Strongly disagree  2 – Disagree  3 – Agree  4 – Strongly agree | 1 – Strongly disagree  2 – Disagree  3 – Agree  4 – Strongly agree | 1 – Strongly disagree  2 – Disagree  3 – Agree  4 – Strongly agree | 1 – Strongly disagree  2 – Disagree  3 – Agree  4 – Strongly agree |
| ECG Holter detection with PACs greater than 10000 PACs per 24h | 1 – Strongly disagree  2 – Disagree  3 – Agree  4 – Strongly agree | 1 – Strongly disagree  2 – Disagree  3 – Agree  4 – Strongly agree | 1 – Strongly disagree  2 – Disagree  3 – Agree  4 – Strongly agree | 1 – Strongly disagree  2 – Disagree  3 – Agree  4 – Strongly agree | 1 – Strongly disagree  2 – Disagree  3 – Agree  4 – Strongly agree |

*Definition of European Staging*

*The European staging system is a tool used to stratify patients with AL amyloidosis.*


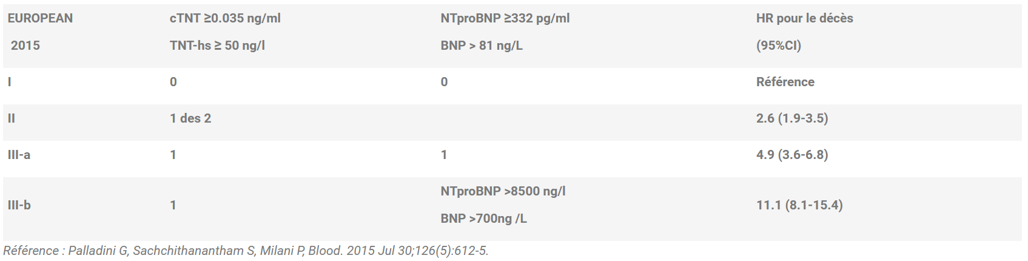


**AF and FLUTTER (AL)**

- **Indicate your level of agreement with the proposed management strategies for atrial fibrillation in a patient with AL for each of the following scenarios:**

|  | **Rhythm control** | **Rate control** |
| --- | --- | --- |
| First episode of symptomatic AF | 1 – Strongly disagree  2 – Disagree  3 – Agree  4 – Strongly agree | 1 – Strongly disagree  2 – Disagree  3 – Agree  4 – Strongly agree |
| First episode of asymptomatic AF | 1 – Strongly disagree  2 – Disagree  3 – Agree  4 – Strongly agree | 1 – Strongly disagree  2 – Disagree  3 – Agree  4 – Strongly agree |
| After recurrence of symptomatic EC, on amiodarone | 1 – Strongly disagree  2 – Disagree  3 – Agree  4 – Strongly agree | 1 – Strongly disagree  2 – Disagree  3 – Agree  4 – Strongly agree |
| After recurrence of asymptomatic EC, on amiodarone | 1 – Strongly disagree  2 – Disagree  3 – Agree  4 – Strongly agree | 1 – Strongly disagree  2 – Disagree  3 – Agree  4 – Strongly agree |

- **Indicate your level of agreement:**

**If the European stage is higher, your management will be more aggressive.**
1 – Strongly disagree 2 – Disagree 3 – Agree 4 – Strongly agree

- **You have chosen a rate control strategy. For a patient with AL presenting with rapid AF and preserved LVEF, indicate your level of agreement with each proposed therapeutic option, and specify whether your management would be more aggressive if the European stage is higher**

|  |  | **If higher European staging** |
| --- | --- | --- |
| Beta-blockers in progressive doses | 1 – Strongly disagree  2 – Disagree  3 – Agree  4 – Strongly agree | 1 – Strongly disagree  2 – Disagree  3 – Agree  4 – Strongly agree |
| Digoxin orally | 1 – Strongly disagree  2 – Disagree  3 – Agree  4 – Strongly agree | 1 – Strongly disagree  2 – Disagree  3 – Agree  4 – Strongly agree |
| Amiodarone to slow heart rate | 1 – Strongly disagree  2 – Disagree  3 – Agree  4 – Strongly agree | 1 – Strongly disagree  2 – Disagree  3 – Agree  4 – Strongly agree |
| Calcium channel blockers | 1 – Strongly disagree  2 – Disagree  3 – Agree  4 – Strongly agree | 1 – Strongly disagree  2 – Disagree  3 – Agree  4 – Strongly agree |
| AVN ablation | 1 – Strongly disagree  2 – Disagree  3 – Agree  4 – Strongly agree | 1 – Strongly disagree  2 – Disagree  3 – Agree  4 – Strongly agree |

- **You have chosen a rhythm control strategy. For a patient with AL presenting with AF and preserved LVEF, indicate your level of agreement with each proposed therapeutic option, and specify whether your management would be more aggressive if the European stage is higher:**

|  |  | **If higher European stage** |
| --- | --- | --- |
| AF ablation | 1 – Strongly disagree  2 – Disagree  3 – Agree  4 – Strongly agree | 1 – Strongly disagree  2 – Disagree  3 – Agree  4 – Strongly agree |
| Amiodarone | 1 – Strongly disagree  2 – Disagree  3 – Agree  4 – Strongly agree | 1 – Strongly disagree  2 – Disagree  3 – Agree  4 – Strongly agree |
| Flecainide | 1 – Strongly disagree  2 – Disagree  3 – Agree  4 – Strongly agree | 1 – Strongly disagree  2 – Disagree  3 – Agree  4 – Strongly agree |
| Sotalol | 1 – Strongly disagree  2 – Disagree  3 – Agree  4 – Strongly agree | 1 – Strongly disagree  2 – Disagree  3 – Agree  4 – Strongly agree |
| Repeated EC | 1 – Strongly disagree  2 – Disagree  3 – Agree  4 – Strongly agree | 1 – Strongly disagree  2 – Disagree  3 – Agree  4 – Strongly agree |

- **You have chosen a cardioversion strategy for a patient with AL, who is effectively anticoagulated. Indicate your level of agreement with performing systematic atrial imaging to check presence of thrombus:**

1 – Strongly disagree 2 – Disagree 3 – Agree 4 – Strongly agree

- **For a patient with AL presenting with typical atrial flutter, indicate your level of agreement with performing first-line ablation:**

1 – Strongly disagree 2 – Disagree 3 – Agree 4 – Strongly agree

**IMPLANTATION (AL)**

- **Indicate your level of agreement with the proposed management approaches for an asymptomatic patient with AL in each of the following scenarios:** *(The different strategies are not mutually exclusive; you are providing your opinion on each strategy independently.)*

|  | **PM/ICD** | **Electrophysiology study** | **Long term monitoring** | **Nothing** |
| --- | --- | --- | --- | --- |
| Narrow QRS and AV block I with PR between 200 and 250 ms | 1 – Strongly disagree  2 – Disagree  3 – Agree  4 – Strongly agree | 1 – Strongly disagree  2 – Disagree  3 – Agree  4 – Strongly agree | 1 – Strongly disagree  2 – Disagree  3 – Agree  4 – Strongly agree | 1 – Strongly disagree  2 – Disagree  3 – Agree  4 – Strongly agree |
| Narrow QRS and AV block I with PR > 250 ms | 1 – Strongly disagree  2 – Disagree  3 – Agree  4 – Strongly agree | 1 – Strongly disagree  2 – Disagree  3 – Agree  4 – Strongly agree | 1 – Strongly disagree  2 – Disagree  3 – Agree  4 – Strongly agree | 1 – Strongly disagree  2 – Disagree  3 – Agree  4 – Strongly agree |
| QRS > 120 ms, right bundle branch block, AV block I with PR between 200 et 250 ms | 1 – Strongly disagree  2 – Disagree  3 – Agree  4 – Strongly agree | 1 – Strongly disagree  2 – Disagree  3 – Agree  4 – Strongly agree | 1 – Strongly disagree  2 – Disagree  3 – Agree  4 – Strongly agree | 1 – Strongly disagree  2 – Disagree  3 – Agree  4 – Strongly agree |
| QRS > 120 ms, left bundle branch block, AV block I with PR between 200 et 250 ms | 1 – Strongly disagree  2 – Disagree  3 – Agree  4 – Strongly agree | 1 – Strongly disagree  2 – Disagree  3 – Agree  4 – Strongly agree | 1 – Strongly disagree  2 – Disagree  3 – Agree  4 – Strongly agree | 1 – Strongly disagree  2 – Disagree  3 – Agree  4 – Strongly agree |
| QRS > 120 ms, right bundle branch block, AV block I with PR > 250 ms | 1 – Strongly disagree  2 – Disagree  3 – Agree  4 – Strongly agree | 1 – Strongly disagree  2 – Disagree  3 – Agree  4 – Strongly agree | 1 – Strongly disagree  2 – Disagree  3 – Agree  4 – Strongly agree | 1 – Strongly disagree  2 – Disagree  3 – Agree  4 – Strongly agree |
| QRS > 120 ms, left bundle branch block, AV block I with PR > 250 ms | 1 – Strongly disagree  2 – Disagree  3 – Agree  4 – Strongly agree | 1 – Strongly disagree  2 – Disagree  3 – Agree  4 – Strongly agree | 1 – Strongly disagree  2 – Disagree  3 – Agree  4 – Strongly agree | 1 – Strongly disagree  2 – Disagree  3 – Agree  4 – Strongly agree |
| Trifascicular block | 1 – Strongly disagree  2 – Disagree  3 – Agree  4 – Strongly agree | 1 – Strongly disagree  2 – Disagree  3 – Agree  4 – Strongly agree | 1 – Strongly disagree  2 – Disagree  3 – Agree  4 – Strongly agree | 1 – Strongly disagree  2 – Disagree  3 – Agree  4 – Strongly agree |
| Evolving conductive disorders | 1 – Strongly disagree  2 – Disagree  3 – Agree  4 – Strongly agree | 1 – Strongly disagree  2 – Disagree  3 – Agree  4 – Strongly agree | 1 – Strongly disagree  2 – Disagree  3 – Agree  4 – Strongly agree | 1 – Strongly disagree  2 – Disagree  3 – Agree  4 – Strongly agree |
| Paroxysmal diurnal transition to AV block II Mobitz 1 | 1 – Strongly disagree  2 – Disagree  3 – Agree  4 – Strongly agree | 1 – Strongly disagree  2 – Disagree  3 – Agree  4 – Strongly agree | 1 – Strongly disagree  2 – Disagree  3 – Agree  4 – Strongly agree | 1 – Strongly disagree  2 – Disagree  3 – Agree  4 – Strongly agree |
| Paroxysmal nocturnal transition to AV block II Mobitz 1 | 1 – Strongly disagree  2 – Disagree  3 – Agree  4 – Strongly agree | 1 – Strongly disagree  2 – Disagree  3 – Agree  4 – Strongly agree | 1 – Strongly disagree  2 – Disagree  3 – Agree  4 – Strongly agree | 1 – Strongly disagree  2 – Disagree  3 – Agree  4 – Strongly agree |
| Tachycardia-bradycardia syndrome | 1 – Strongly disagree  2 – Disagree  3 – Agree  4 – Strongly agree | 1 – Strongly disagree  2 – Disagree  3 – Agree  4 – Strongly agree | 1 – Strongly disagree  2 – Disagree  3 – Agree  4 – Strongly agree | 1 – Strongly disagree  2 – Disagree  3 – Agree  4 – Strongly agree |
| Sinus node dysfunction with diurnal pauses pause between 3 and 6 sec | 1 – Strongly disagree  2 – Disagree  3 – Agree  4 – Strongly agree | 1 – Strongly disagree  2 – Disagree  3 – Agree  4 – Strongly agree | 1 – Strongly disagree  2 – Disagree  3 – Agree  4 – Strongly agree | 1 – Strongly disagree  2 – Disagree  3 – Agree  4 – Strongly agree |

*Definitions*

***Trifascicular block****: A trifascicular block is an electrical conduction problem in the heart, specifically involving all three fascicles of the bundle branches that transmit electrical signals from the atrioventricular node to the ventricles.*

***Tachycardia-bradycardia syndrome*** *is a heart rhythm disorder that affects the atria of the heart. It is characterized by alterations in atrial tissue, which can lead to irregular heart rhythms of varying frequency. Symptoms may include palpitations, dizziness, fatigue, chest pain, and heart failure.*

- **Indicate your level of agreement for each criterion. For a patient with AL who has an indication for pacing, the following criteria influence your decision to implant a defibrillator**

**LVEF**
1 – Strongly disagree 2 – Disagree 3 – Agree 4 – Strongly agree

**GLS**
1 – Strongly disagree 2 – Disagree 3 – Agree 4 – Strongly agree

**NSVT**
1 – Strongly disagree 2 – Disagree 3 – Agree 4 – Strongly agree

- **Indicate your level of agreement for each criterion. For a patient with AL who does not have an indication for pacing, the following criteria influence your decision to implant a defibrillator:**

**LVEF**
1 – Strongly disagree 2 – Disagree 3 – Agree 4 – Strongly agree

**GLS**
1 – Strongly disagree 2 – Disagree 3 – Agree 4 – Strongly agree

**NSVT**
1 – Strongly disagree 2 – Disagree 3 – Agree 4 – Strongly agree

- **Indicate your level of agreement. For a patient with AL who has an indication for ventricular pacing.**

**The LVEF will influence a decision for CRT:**

1 – Strongly disagree 2 – Disagree 3 – Agree 4 – Strongly agree

**A cut-off at 50% seems appropriate:**

1 – Strongly disagree 2 – Disagree 3 – Agree 4 – Strongly agree

**The Global Longitudinal Strain will influence a decision for CRT:**
1 – Strongly disagree 2 – Disagree 3 – Agree 4 – Strongly agree

**A cut-off at 14% seems appropriate:**

1 – Strongly disagree 2 – Disagree 3 – Agree 4 – Strongly agree

**The expected percentage of pacing will influence a decision for CRT:**
1 – Strongly disagree 2 – Disagree 3 – Agree 4 – Strongly agree

**SATISFACTION
How did you find this questionnaire?**


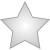
 
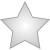
 
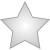
 
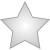
 
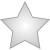


**Do you have any remarks or comments about the questionnaire you just completed?**

**CONCLUSION**

**This questionnaire is now complete.**

**Thank you for your participation.**

**Supplemental Document 2. Delphi questionnaire (French original)**

**INTRODUCTION**

Bienvenue !

Aujourd’hui, nous avons un nouveau questionnaire à vous proposer, intitulé DELPHI prise en charge rythmologique.

Celui-ci est diffusé sur notre site dans le cadre d'une étude portant sur la prise en charge rythmologique des patients atteints d’amyloses cardiaques, et ayant pour objectifs de mieux connaitre vos habitudes, pratiques et avis sur ce sujet.

Vos réponses seront traitées uniquement à des fins statistiques.

Nous avons estimé le temps nécessaire pour répondre au questionnaire à 30 minutes environ. Nous vous conseillons de répondre en une seule fois au questionnaire. Vos réponses sont sauvegardées pendant un certain temps mais il faut pour cela utiliser le même ordinateur et le même navigateur internet.

Merci d’avance pour votre participation.

- **Veuillez indiquer le code qui vous a été attribué :**

Code :

- **Acceptez-vous de renoncer à la confidentialité liée à l’événement indésirable et de transmettre vos coordonnées à notre client aux fins de la déclaration d’événements indésirables ?** *(Une seule réponse possible)*

Oui

Non

- **Êtes-vous ?** *(Une seule réponse possible)*

Cardiologue

Rythmologue

- **Dans quel département ?**

Département :

- **Prenez-vous en charge des patients ATTRwt ou ATTRv CM ?** *(Une seule réponse possible)*

Oui

Non

- **Si oui en moyenne, combien de patients prenez-vous en charge ?** *(Une seule réponse possible)*

Entre 0 et 10

Entre 10 et 50

Supérieur à 50

- **Prenez-vous en charge des patients amylose AL ?** *(Une seule réponse possible)*

Oui

Non

- **Si oui en moyenne, combien de patients prenez-vous en charge ?** *(Une seule réponse possible)*

Entre 0 et 10

Entre 10 et 50

Supérieur à 50

- **Combien d'années d'expériences avez-vous dans la prise en charge de ces patients ?** *(Une seule réponse possible)*

Moins de 5 ans

Entre 5 et 10 ans

Entre 10 et 15 ans

Plus de 15 ans

**ANTICOAGULATION (ATTR)**

- **Indiquez votre degré d'accord dans les prises en charge proposées devant un patient ATTRwt ou ATTRv CM en rythme sinusal pour chacune des situations suivantes, et indiquez dans les deux dernières colonnes si votre prise en charge serait plus agressive en cas de stade NAC plus élevé ou de présence de salves :** *(Les différentes stratégies ne sont pas mutuellement exclusives, vous donnez votre avis pour chaque stratégie indépendamment)

  Stratégies de prise en charge*

|  | **Holters de 24h réguliers (tous les 6 à 12 mois)** | **Enregistrement de longue durée** | **Anticoagulants** | **Si stade NAC plus élevé** | **Si présence de salves** |
| --- | --- | --- | --- | --- | --- |
| Antécédents d'AVC avec territoire sur imagerie cérébrale compatible avec événement cardio-embolique | 1 – Pas du tout d’accord  2 – Plutôt pas d’accord  3 – Plutôt d’accord  4 – Tout à fait d’accord | 1 – Pas du tout d’accord  2 – Plutôt pas d’accord  3 – Plutôt d’accord  4 – Tout à fait d’accord | 1 – Pas du tout d’accord  2 – Plutôt pas d’accord  3 – Plutôt d’accord  4 – Tout à fait d’accord | 1 – Pas du tout d’accord  2 – Plutôt pas d’accord  3 – Plutôt d’accord  4 – Tout à fait d’accord |  |
| Antécédents d'AIT récent (moins de 1 an) | 1 – Pas du tout d’accord  2 – Plutôt pas d’accord  3 – Plutôt d’accord  4 – Tout à fait d’accord | 1 – Pas du tout d’accord  2 – Plutôt pas d’accord  3 – Plutôt d’accord  4 – Tout à fait d’accord | 1 – Pas du tout d’accord  2 – Plutôt pas d’accord  3 – Plutôt d’accord  4 – Tout à fait d’accord | 1 – Pas du tout d’accord  2 – Plutôt pas d’accord  3 – Plutôt d’accord  4 – Tout à fait d’accord |  |
| Dépistage de fibrillation auriculaire sur dispositif cardiaque implantable < 6 min |  |  | 1 – Pas du tout d’accord  2 – Plutôt pas d’accord  3 – Plutôt d’accord  4 – Tout à fait d’accord | 1 – Pas du tout d’accord  2 – Plutôt pas d’accord  3 – Plutôt d’accord  4 – Tout à fait d’accord |  |
| Profil mitral avec onde E exclusive | 1 – Pas du tout d’accord  2 – Plutôt pas d’accord  3 – Plutôt d’accord  4 – Tout à fait d’accord | 1 – Pas du tout d’accord  2 – Plutôt pas d’accord  3 – Plutôt d’accord  4 – Tout à fait d’accord | 1 – Pas du tout d’accord  2 – Plutôt pas d’accord  3 – Plutôt d’accord  4 – Tout à fait d’accord | 1 – Pas du tout d’accord  2 – Plutôt pas d’accord  3 – Plutôt d’accord  4 – Tout à fait d’accord |  |
| Profil mitral restrictif | 1 – Pas du tout d’accord  2 – Plutôt pas d’accord  3 – Plutôt d’accord  4 – Tout à fait d’accord | 1 – Pas du tout d’accord  2 – Plutôt pas d’accord  3 – Plutôt d’accord  4 – Tout à fait d’accord | 1 – Pas du tout d’accord  2 – Plutôt pas d’accord  3 – Plutôt d’accord  4 – Tout à fait d’accord | 1 – Pas du tout d’accord  2 – Plutôt pas d’accord  3 – Plutôt d’accord  4 – Tout à fait d’accord |  |
| Score CHA2DS2-VASc ≥ 3, sans autre facteur de risque thromboembolique (hormis amylose) | 1 – Pas du tout d’accord  2 – Plutôt pas d’accord  3 – Plutôt d’accord  4 – Tout à fait d’accord | 1 – Pas du tout d’accord  2 – Plutôt pas d’accord  3 – Plutôt d’accord  4 – Tout à fait d’accord | 1 – Pas du tout d’accord  2 – Plutôt pas d’accord  3 – Plutôt d’accord  4 – Tout à fait d’accord | 1 – Pas du tout d’accord  2 – Plutôt pas d’accord  3 – Plutôt d’accord  4 – Tout à fait d’accord |  |
| Fraction d'éjection du ventricule gauche ≤ 50 % | 1 – Pas du tout d’accord  2 – Plutôt pas d’accord  3 – Plutôt d’accord  4 – Tout à fait d’accord | 1 – Pas du tout d’accord  2 – Plutôt pas d’accord  3 – Plutôt d’accord  4 – Tout à fait d’accord | 1 – Pas du tout d’accord  2 – Plutôt pas d’accord  3 – Plutôt d’accord  4 – Tout à fait d’accord | 1 – Pas du tout d’accord  2 – Plutôt pas d’accord  3 – Plutôt d’accord  4 – Tout à fait d’accord |  |
| Fraction d'éjection du ventricule gauche ≤ 30 % | 1 – Pas du tout d’accord  2 – Plutôt pas d’accord  3 – Plutôt d’accord  4 – Tout à fait d’accord | 1 – Pas du tout d’accord  2 – Plutôt pas d’accord  3 – Plutôt d’accord  4 – Tout à fait d’accord | 1 – Pas du tout d’accord  2 – Plutôt pas d’accord  3 – Plutôt d’accord  4 – Tout à fait d’accord | 1 – Pas du tout d’accord  2 – Plutôt pas d’accord  3 – Plutôt d’accord  4 – Tout à fait d’accord |  |
| Dépistage sur un holter ECG de suivi d'une hyperexcitabilité atriale avec 500 à 1000 ESA par 24h | 1 – Pas du tout d’accord  2 – Plutôt pas d’accord  3 – Plutôt d’accord  4 – Tout à fait d’accord | 1 – Pas du tout d’accord  2 – Plutôt pas d’accord  3 – Plutôt d’accord  4 – Tout à fait d’accord | 1 – Pas du tout d’accord  2 – Plutôt pas d’accord  3 – Plutôt d’accord  4 – Tout à fait d’accord | 1 – Pas du tout d’accord  2 – Plutôt pas d’accord  3 – Plutôt d’accord  4 – Tout à fait d’accord | 1 – Pas du tout d’accord  2 – Plutôt pas d’accord  3 – Plutôt d’accord  4 – Tout à fait d’accord |
| Dépistage sur un holter ECG de suivi d'une hyperexcitabilité atriale avec 1000 à 10000 ESA par 24h | 1 – Pas du tout d’accord  2 – Plutôt pas d’accord  3 – Plutôt d’accord  4 – Tout à fait d’accord | 1 – Pas du tout d’accord  2 – Plutôt pas d’accord  3 – Plutôt d’accord  4 – Tout à fait d’accord | 1 – Pas du tout d’accord  2 – Plutôt pas d’accord  3 – Plutôt d’accord  4 – Tout à fait d’accord | 1 – Pas du tout d’accord  2 – Plutôt pas d’accord  3 – Plutôt d’accord  4 – Tout à fait d’accord | 1 – Pas du tout d’accord  2 – Plutôt pas d’accord  3 – Plutôt d’accord  4 – Tout à fait d’accord |
| Dépistage sur un holter ECG de suivi d'une hyperexcitabilité atriale supérieure à 10000 ESA par 24h | 1 – Pas du tout d’accord  2 – Plutôt pas d’accord  3 – Plutôt d’accord  4 – Tout à fait d’accord | 1 – Pas du tout d’accord  2 – Plutôt pas d’accord  3 – Plutôt d’accord  4 – Tout à fait d’accord | 1 – Pas du tout d’accord  2 – Plutôt pas d’accord  3 – Plutôt d’accord  4 – Tout à fait d’accord | 1 – Pas du tout d’accord  2 – Plutôt pas d’accord  3 – Plutôt d’accord  4 – Tout à fait d’accord | 1 – Pas du tout d’accord  2 – Plutôt pas d’accord  3 – Plutôt d’accord  4 – Tout à fait d’accord |

*Définition de la NAC*

*Le score « NAC » (pour National Amyloïdosis Center) développé par Gillmore et al. stratifie les patients atteints d’amylose ATTR dans 3 groupes de survie selon l’élévation du NT-proBNP >3000 ng/ L et la diminution du débit de filtration glomérulaire (DFG) estimé par la formule du MDRD < 45 mL/min/1,73m2.*


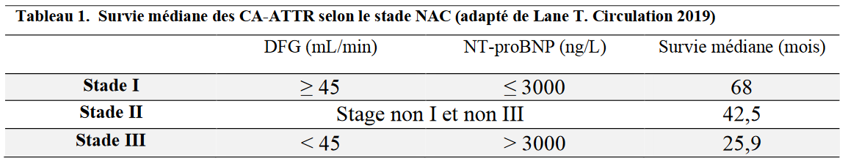


**FA ET FLUTTER (ATTR)**

- **Indiquez votre degré d'accord dans les prises en charge de la fibrillation atriale proposées devant un patient ATTRwt ou ATTRv CM pour chacune des situations suivantes :**

|  | **Contrôle du rythme** | **Contrôle de la fréquence** |
| --- | --- | --- |
| Premier épisode de FA symptomatique | 1 – Pas du tout d’accord  2 – Plutôt pas d’accord  3 – Plutôt d’accord  4 – Tout à fait d’accord | 1 – Pas du tout d’accord  2 – Plutôt pas d’accord  3 – Plutôt d’accord  4 – Tout à fait d’accord |
| Premier épisode de FA asymptomatique | 1 – Pas du tout d’accord  2 – Plutôt pas d’accord  3 – Plutôt d’accord  4 – Tout à fait d’accord | 1 – Pas du tout d’accord  2 – Plutôt pas d’accord  3 – Plutôt d’accord  4 – Tout à fait d’accord |
| Après récidive post CEE symptomatique, et sous amiodarone | 1 – Pas du tout d’accord  2 – Plutôt pas d’accord  3 – Plutôt d’accord  4 – Tout à fait d’accord | 1 – Pas du tout d’accord  2 – Plutôt pas d’accord  3 – Plutôt d’accord  4 – Tout à fait d’accord |
| Après récidive post CEE asymptomatique, et sous amiodarone | 1 – Pas du tout d’accord  2 – Plutôt pas d’accord  3 – Plutôt d’accord  4 – Tout à fait d’accord | 1 – Pas du tout d’accord  2 – Plutôt pas d’accord  3 – Plutôt d’accord  4 – Tout à fait d’accord |

- **Indiquez votre degré d'accord :**

**Si le stade NAC est plus élevé, votre prise en charge sera plus agressive**
1 – Pas du tout d’accord 2 – Plutôt pas d’accord 3 – Plutôt d’accord 4 – Tout à fait d’accord

- **Vous avez choisi une stratégie de contrôle de la fréquence. Devant un patient ATTRwt ou ATTRv CM présentant une FA rapide avec FEVG préservée, indiquez votre degré d'accord avec chacune des thérapeutiques proposées, puis si le stade NAC est plus élevé :**

|  |  | **Si stade NAC plus élevé** |
| --- | --- | --- |
| Bétabloquants à doses progressives | 1 – Pas du tout d’accord  2 – Plutôt pas d’accord  3 – Plutôt d’accord  4 – Tout à fait d’accord | 1 – Pas du tout d’accord  2 – Plutôt pas d’accord  3 – Plutôt d’accord  4 – Tout à fait d’accord |
| Digoxine per os | 1 – Pas du tout d’accord  2 – Plutôt pas d’accord  3 – Plutôt d’accord  4 – Tout à fait d’accord | 1 – Pas du tout d’accord  2 – Plutôt pas d’accord  3 – Plutôt d’accord  4 – Tout à fait d’accord |
| Amiodarone à visée de ralentissement de la FC | 1 – Pas du tout d’accord  2 – Plutôt pas d’accord  3 – Plutôt d’accord  4 – Tout à fait d’accord | 1 – Pas du tout d’accord  2 – Plutôt pas d’accord  3 – Plutôt d’accord  4 – Tout à fait d’accord |
| Inhibiteurs calciques | 1 – Pas du tout d’accord  2 – Plutôt pas d’accord  3 – Plutôt d’accord  4 – Tout à fait d’accord | 1 – Pas du tout d’accord  2 – Plutôt pas d’accord  3 – Plutôt d’accord  4 – Tout à fait d’accord |
| Ablation NAV | 1 – Pas du tout d’accord  2 – Plutôt pas d’accord  3 – Plutôt d’accord  4 – Tout à fait d’accord | 1 – Pas du tout d’accord  2 – Plutôt pas d’accord  3 – Plutôt d’accord  4 – Tout à fait d’accord |

- **Vous avez choisi une stratégie de contrôle du rythme. Devant un patient ATTRwt ou ATTRv CM présentant une FA avec FEVG préservée, indiquez votre degré d'accord avec chacune des thérapeutiques proposées, puis si le stade NAC est plus élevé :**

|  |  | **Si stade NAC plus élevé** |
| --- | --- | --- |
| Ablation de FA | 1 – Pas du tout d’accord  2 – Plutôt pas d’accord  3 – Plutôt d’accord  4 – Tout à fait d’accord | 1 – Pas du tout d’accord  2 – Plutôt pas d’accord  3 – Plutôt d’accord  4 – Tout à fait d’accord |
| Amiodarone | 1 – Pas du tout d’accord  2 – Plutôt pas d’accord  3 – Plutôt d’accord  4 – Tout à fait d’accord | 1 – Pas du tout d’accord  2 – Plutôt pas d’accord  3 – Plutôt d’accord  4 – Tout à fait d’accord |
| Flécaine | 1 – Pas du tout d’accord  2 – Plutôt pas d’accord  3 – Plutôt d’accord  4 – Tout à fait d’accord | 1 – Pas du tout d’accord  2 – Plutôt pas d’accord  3 – Plutôt d’accord  4 – Tout à fait d’accord |
| Sotalol | 1 – Pas du tout d’accord  2 – Plutôt pas d’accord  3 – Plutôt d’accord  4 – Tout à fait d’accord | 1 – Pas du tout d’accord  2 – Plutôt pas d’accord  3 – Plutôt d’accord  4 – Tout à fait d’accord |
| CEE répétés | 1 – Pas du tout d’accord  2 – Plutôt pas d’accord  3 – Plutôt d’accord  4 – Tout à fait d’accord | 1 – Pas du tout d’accord  2 – Plutôt pas d’accord  3 – Plutôt d’accord  4 – Tout à fait d’accord |

- **Vous avez choisi une stratégie de cardioversion chez un patient ATTRwt ou ATTRv CM, anticoagulé de façon efficace. Indiquez votre degré d'accord avec la réalisation d'une imagerie systématique de l'auricule pour vérifier la présence d'un thrombus :**

1 – Pas du tout d’accord 2 – Plutôt pas d’accord 3 – Plutôt d’accord 4 – Tout à fait d’accord

- **Devant un patient ATTRwt ou ATTRv CM présentant un flutter atrial commun, indiquez votre degré d'accord avec la réalisation d'une ablation en première intention :**

1 – Pas du tout d’accord 2 – Plutôt pas d’accord 3 – Plutôt d’accord 4 – Tout à fait d’accord

**IMPLANTATION (ATTR)**

- **Indiquez votre degré d'accord vis-à-vis des prises en charge proposées devant un patient ATTRwt ou ATTRv CM asymptomatique pour chacune des situations suivantes :***(Les différentes stratégies ne sont pas mutuellement exclusives, vous donnez votre avis pour chaque stratégie indépendamment)*

|  | **Pacemaker/Défibrillateur automatique implantable** | **Examen électrophysiologique** | **Enregistrement de longue durée** | **Rien** |
| --- | --- | --- | --- | --- |
| QRS fin et BAV I avec PR entre 200 et 250 ms | 1 – Pas du tout d’accord  2 – Plutôt pas d’accord  3 – Plutôt d’accord  4 – Tout à fait d’accord | 1 – Pas du tout d’accord  2 – Plutôt pas d’accord  3 – Plutôt d’accord  4 – Tout à fait d’accord | 1 – Pas du tout d’accord  2 – Plutôt pas d’accord  3 – Plutôt d’accord  4 – Tout à fait d’accord | 1 – Pas du tout d’accord  2 – Plutôt pas d’accord  3 – Plutôt d’accord  4 – Tout à fait d’accord |
| QRS fin et BAV I avec PR > 250 ms | 1 – Pas du tout d’accord  2 – Plutôt pas d’accord  3 – Plutôt d’accord  4 – Tout à fait d’accord | 1 – Pas du tout d’accord  2 – Plutôt pas d’accord  3 – Plutôt d’accord  4 – Tout à fait d’accord | 1 – Pas du tout d’accord  2 – Plutôt pas d’accord  3 – Plutôt d’accord  4 – Tout à fait d’accord | 1 – Pas du tout d’accord  2 – Plutôt pas d’accord  3 – Plutôt d’accord  4 – Tout à fait d’accord |
| QRS > 120 ms, bloc de branche droit, BAV I avec PR entre 200 et 250 ms | 1 – Pas du tout d’accord  2 – Plutôt pas d’accord  3 – Plutôt d’accord  4 – Tout à fait d’accord | 1 – Pas du tout d’accord  2 – Plutôt pas d’accord  3 – Plutôt d’accord  4 – Tout à fait d’accord | 1 – Pas du tout d’accord  2 – Plutôt pas d’accord  3 – Plutôt d’accord  4 – Tout à fait d’accord | 1 – Pas du tout d’accord  2 – Plutôt pas d’accord  3 – Plutôt d’accord  4 – Tout à fait d’accord |
| QRS > 120 ms, bloc de branche gauche, BAV I avec PR entre 200 et 250 ms | 1 – Pas du tout d’accord  2 – Plutôt pas d’accord  3 – Plutôt d’accord  4 – Tout à fait d’accord | 1 – Pas du tout d’accord  2 – Plutôt pas d’accord  3 – Plutôt d’accord  4 – Tout à fait d’accord | 1 – Pas du tout d’accord  2 – Plutôt pas d’accord  3 – Plutôt d’accord  4 – Tout à fait d’accord | 1 – Pas du tout d’accord  2 – Plutôt pas d’accord  3 – Plutôt d’accord  4 – Tout à fait d’accord |
| QRS > 120 ms, bloc de branche droit, BAV I avec PR > 250 ms | 1 – Pas du tout d’accord  2 – Plutôt pas d’accord  3 – Plutôt d’accord  4 – Tout à fait d’accord | 1 – Pas du tout d’accord  2 – Plutôt pas d’accord  3 – Plutôt d’accord  4 – Tout à fait d’accord | 1 – Pas du tout d’accord  2 – Plutôt pas d’accord  3 – Plutôt d’accord  4 – Tout à fait d’accord | 1 – Pas du tout d’accord  2 – Plutôt pas d’accord  3 – Plutôt d’accord  4 – Tout à fait d’accord |
| QRS > 120 ms, bloc de branche gauche, BAV I avec PR > 250 ms | 1 – Pas du tout d’accord  2 – Plutôt pas d’accord  3 – Plutôt d’accord  4 – Tout à fait d’accord | 1 – Pas du tout d’accord  2 – Plutôt pas d’accord  3 – Plutôt d’accord  4 – Tout à fait d’accord | 1 – Pas du tout d’accord  2 – Plutôt pas d’accord  3 – Plutôt d’accord  4 – Tout à fait d’accord | 1 – Pas du tout d’accord  2 – Plutôt pas d’accord  3 – Plutôt d’accord  4 – Tout à fait d’accord |
| Bloc trifasciculaire  Définition | 1 – Pas du tout d’accord  2 – Plutôt pas d’accord  3 – Plutôt d’accord  4 – Tout à fait d’accord | 1 – Pas du tout d’accord  2 – Plutôt pas d’accord  3 – Plutôt d’accord  4 – Tout à fait d’accord | 1 – Pas du tout d’accord  2 – Plutôt pas d’accord  3 – Plutôt d’accord  4 – Tout à fait d’accord | 1 – Pas du tout d’accord  2 – Plutôt pas d’accord  3 – Plutôt d’accord  4 – Tout à fait d’accord |
| Troubles conductifs évolutifs | 1 – Pas du tout d’accord  2 – Plutôt pas d’accord  3 – Plutôt d’accord  4 – Tout à fait d’accord | 1 – Pas du tout d’accord  2 – Plutôt pas d’accord  3 – Plutôt d’accord  4 – Tout à fait d’accord | 1 – Pas du tout d’accord  2 – Plutôt pas d’accord  3 – Plutôt d’accord  4 – Tout à fait d’accord | 1 – Pas du tout d’accord  2 – Plutôt pas d’accord  3 – Plutôt d’accord  4 – Tout à fait d’accord |
| Passage paroxystique en BAV II Mobitz 1 | 1 – Pas du tout d’accord  2 – Plutôt pas d’accord  3 – Plutôt d’accord  4 – Tout à fait d’accord | 1 – Pas du tout d’accord  2 – Plutôt pas d’accord  3 – Plutôt d’accord  4 – Tout à fait d’accord | 1 – Pas du tout d’accord  2 – Plutôt pas d’accord  3 – Plutôt d’accord  4 – Tout à fait d’accord | 1 – Pas du tout d’accord  2 – Plutôt pas d’accord  3 – Plutôt d’accord  4 – Tout à fait d’accord |
| Maladie Rythmique Atriale  Définition | 1 – Pas du tout d’accord  2 – Plutôt pas d’accord  3 – Plutôt d’accord  4 – Tout à fait d’accord | 1 – Pas du tout d’accord  2 – Plutôt pas d’accord  3 – Plutôt d’accord  4 – Tout à fait d’accord | 1 – Pas du tout d’accord  2 – Plutôt pas d’accord  3 – Plutôt d’accord  4 – Tout à fait d’accord | 1 – Pas du tout d’accord  2 – Plutôt pas d’accord  3 – Plutôt d’accord  4 – Tout à fait d’accord |
| Pause sinusale entre 3 et 6 sec. diurne | 1 – Pas du tout d’accord  2 – Plutôt pas d’accord  3 – Plutôt d’accord  4 – Tout à fait d’accord | 1 – Pas du tout d’accord  2 – Plutôt pas d’accord  3 – Plutôt d’accord  4 – Tout à fait d’accord | 1 – Pas du tout d’accord  2 – Plutôt pas d’accord  3 – Plutôt d’accord  4 – Tout à fait d’accord | 1 – Pas du tout d’accord  2 – Plutôt pas d’accord  3 – Plutôt d’accord  4 – Tout à fait d’accord |

*Définitions*

*Un bloc trifasciculaire est un problème de conduction électrique du cœur, plus précisément des trois faisceaux des branches du faisceau qui transportent les signaux électriques du nœud auriculo-ventriculaire aux ventricules.*

*Une maladie rythmique atriale est un trouble du rythme cardiaque qui affecte les oreillettes du cœur. Cette maladie est caractérisée par une altération du tissu de l'oreillette, qui peut entraîner des anomalies du rythme cardiaque à fréquence variable. Les symptômes de la maladie rythmique atriale peuvent inclure des palpitations, des étourdissements, une fatigue, une douleur thoracique et une insuffisance cardiaque.*

- **Indiquez votre degré d'accord pour chaque critère. Face à un patient ATTRwt ou ATTRv CM ayant une indication de stimulation, les critères suivants influencent votre décision de mettre en place un défibrillateur :**

**La FEVG**
1 – Pas du tout d’accord 2 – Plutôt pas d’accord 3 – Plutôt d’accord 4 – Tout à fait d’accord

**Le Strain Global Longitudinal**
1 – Pas du tout d’accord 2 – Plutôt pas d’accord 3 – Plutôt d’accord 4 – Tout à fait d’accord

**Les TVNS**
1 – Pas du tout d’accord 2 – Plutôt pas d’accord 3 – Plutôt d’accord 4 – Tout à fait d’accord

- **Indiquez votre degré d'accord pour chaque critère. Face à un patient ATTRwt ou ATTRv CM n'ayant pas une indication de stimulation, les critères suivants influencent votre décision de mettre en place un défibrillateur :**

**La FEVG**
1 – Pas du tout d’accord 2 – Plutôt pas d’accord 3 – Plutôt d’accord 4 – Tout à fait d’accord

**Le Strain Global Longitudinal**
1 – Pas du tout d’accord 2 – Plutôt pas d’accord 3 – Plutôt d’accord 4 – Tout à fait d’accord

**Les TVNS**
1 – Pas du tout d’accord 2 – Plutôt pas d’accord 3 – Plutôt d’accord 4 – Tout à fait d’accord

**Le caractère héréditaire de l'ATTR**
1 – Pas du tout d’accord 2 – Plutôt pas d’accord 3 – Plutôt d’accord 4 – Tout à fait d’accord

- **Indiquez votre degré d'accord. Face à un patient ATTRwt ou ATTRv CM ayant une indication de stimulation ventriculaire :**

**La FEVG va influencer une décision de CRT**
1 – Pas du tout d’accord 2 – Plutôt pas d’accord 3 – Plutôt d’accord 4 – Tout à fait d’accord

**Un cut-off à 50% vous semble adapté**

1 – Pas du tout d’accord 2 – Plutôt pas d’accord 3 – Plutôt d’accord 4 – Tout à fait d’accord

**Le Strain Global Longitudinal va influencer une décision de CRT**
1 – Pas du tout d’accord 2 – Plutôt pas d’accord 3 – Plutôt d’accord 4 – Tout à fait d’accord

**Un cut-off à 14% vous semble adapté**

1 – Pas du tout d’accord 2 – Plutôt pas d’accord 3 – Plutôt d’accord 4 – Tout à fait d’accord

**Le pourcentage de stimulation attendu va influencer une décision de CRT**
1 – Pas du tout d’accord 2 – Plutôt pas d’accord 3 – Plutôt d’accord 4 – Tout à fait d’accord

**ANTICOAGULATION (AL)**

- **Indiquez votre degré d'accord dans les prises en charge proposées devant un patient amylose AL en rythme sinusal pour chacune des situations suivantes, et indiquez dans les deux dernières colonnes si votre prise en charge serait plus agressive en cas de score de l'European Staging plus élevé ou de présence de salves :***(Les différentes stratégies de prise en charge ne sont pas mutuellement exclusives, vous donnez votre avis pour chaque stratégie indépendamment).

  Stratégies de prise en charge*

|  | **Holters de 24h réguliers (tous les 6 à 12 mois)** | **Enregistrement de longue durée** | **Anticoagulants** | **Si European Staging plus élevé** | **Si présence de salves** |
| --- | --- | --- | --- | --- | --- |
| Antécédents d'AVC avec territoire sur imagerie cérébrale compatible avec événement cardio-embolique | 1 – Pas du tout d’accord  2 – Plutôt pas d’accord  3 – Plutôt d’accord  4 – Tout à fait d’accord | 1 – Pas du tout d’accord  2 – Plutôt pas d’accord  3 – Plutôt d’accord  4 – Tout à fait d’accord | 1 – Pas du tout d’accord  2 – Plutôt pas d’accord  3 – Plutôt d’accord  4 – Tout à fait d’accord | 1 – Pas du tout d’accord  2 – Plutôt pas d’accord  3 – Plutôt d’accord  4 – Tout à fait d’accord |  |
| Antécédents d'AIT récent (moins de 1 an) | 1 – Pas du tout d’accord  2 – Plutôt pas d’accord  3 – Plutôt d’accord  4 – Tout à fait d’accord | 1 – Pas du tout d’accord  2 – Plutôt pas d’accord  3 – Plutôt d’accord  4 – Tout à fait d’accord | 1 – Pas du tout d’accord  2 – Plutôt pas d’accord  3 – Plutôt d’accord  4 – Tout à fait d’accord | 1 – Pas du tout d’accord  2 – Plutôt pas d’accord  3 – Plutôt d’accord  4 – Tout à fait d’accord |  |
| Dépistage de fibrillation auriculaire sur dispositif cardiaque implantable < 6 min |  |  | 1 – Pas du tout d’accord  2 – Plutôt pas d’accord  3 – Plutôt d’accord  4 – Tout à fait d’accord | 1 – Pas du tout d’accord  2 – Plutôt pas d’accord  3 – Plutôt d’accord  4 – Tout à fait d’accord |  |
| Profil mitral avec onde E exclusive | 1 – Pas du tout d’accord  2 – Plutôt pas d’accord  3 – Plutôt d’accord  4 – Tout à fait d’accord | 1 – Pas du tout d’accord  2 – Plutôt pas d’accord  3 – Plutôt d’accord  4 – Tout à fait d’accord | 1 – Pas du tout d’accord  2 – Plutôt pas d’accord  3 – Plutôt d’accord  4 – Tout à fait d’accord | 1 – Pas du tout d’accord  2 – Plutôt pas d’accord  3 – Plutôt d’accord  4 – Tout à fait d’accord |  |
| Profil mitral restrictif | 1 – Pas du tout d’accord  2 – Plutôt pas d’accord  3 – Plutôt d’accord  4 – Tout à fait d’accord | 1 – Pas du tout d’accord  2 – Plutôt pas d’accord  3 – Plutôt d’accord  4 – Tout à fait d’accord | 1 – Pas du tout d’accord  2 – Plutôt pas d’accord  3 – Plutôt d’accord  4 – Tout à fait d’accord | 1 – Pas du tout d’accord  2 – Plutôt pas d’accord  3 – Plutôt d’accord  4 – Tout à fait d’accord |  |
| Score CHA2DS2-VASc ≥ 3 , sans autre facteur de risque thromboembolique (hormis amylose) | 1 – Pas du tout d’accord  2 – Plutôt pas d’accord  3 – Plutôt d’accord  4 – Tout à fait d’accord | 1 – Pas du tout d’accord  2 – Plutôt pas d’accord  3 – Plutôt d’accord  4 – Tout à fait d’accord | 1 – Pas du tout d’accord  2 – Plutôt pas d’accord  3 – Plutôt d’accord  4 – Tout à fait d’accord | 1 – Pas du tout d’accord  2 – Plutôt pas d’accord  3 – Plutôt d’accord  4 – Tout à fait d’accord |  |
| Fraction d'éjection du ventricule gauche ≤ 50 % | 1 – Pas du tout d’accord  2 – Plutôt pas d’accord  3 – Plutôt d’accord  4 – Tout à fait d’accord | 1 – Pas du tout d’accord  2 – Plutôt pas d’accord  3 – Plutôt d’accord  4 – Tout à fait d’accord | 1 – Pas du tout d’accord  2 – Plutôt pas d’accord  3 – Plutôt d’accord  4 – Tout à fait d’accord | 1 – Pas du tout d’accord  2 – Plutôt pas d’accord  3 – Plutôt d’accord  4 – Tout à fait d’accord |  |
| Fraction d'éjection du ventricule gauche ≤ 30 % | 1 – Pas du tout d’accord  2 – Plutôt pas d’accord  3 – Plutôt d’accord  4 – Tout à fait d’accord | 1 – Pas du tout d’accord  2 – Plutôt pas d’accord  3 – Plutôt d’accord  4 – Tout à fait d’accord | 1 – Pas du tout d’accord  2 – Plutôt pas d’accord  3 – Plutôt d’accord  4 – Tout à fait d’accord | 1 – Pas du tout d’accord  2 – Plutôt pas d’accord  3 – Plutôt d’accord  4 – Tout à fait d’accord |  |
| Dépistage sur un holter ECG de suivi d'une hyperexcitabilité atriale avec 500 à 1000 ESA par 24h | 1 – Pas du tout d’accord  2 – Plutôt pas d’accord  3 – Plutôt d’accord  4 – Tout à fait d’accord | 1 – Pas du tout d’accord  2 – Plutôt pas d’accord  3 – Plutôt d’accord  4 – Tout à fait d’accord | 1 – Pas du tout d’accord  2 – Plutôt pas d’accord  3 – Plutôt d’accord  4 – Tout à fait d’accord | 1 – Pas du tout d’accord  2 – Plutôt pas d’accord  3 – Plutôt d’accord  4 – Tout à fait d’accord | 1 – Pas du tout d’accord  2 – Plutôt pas d’accord  3 – Plutôt d’accord  4 – Tout à fait d’accord |
| Dépistage sur un holter ECG de suivi d'une hyperexcitabilité atriale avec 1000 à 10000 ESA par 24h | 1 – Pas du tout d’accord  2 – Plutôt pas d’accord  3 – Plutôt d’accord  4 – Tout à fait d’accord | 1 – Pas du tout d’accord  2 – Plutôt pas d’accord  3 – Plutôt d’accord  4 – Tout à fait d’accord | 1 – Pas du tout d’accord  2 – Plutôt pas d’accord  3 – Plutôt d’accord  4 – Tout à fait d’accord | 1 – Pas du tout d’accord  2 – Plutôt pas d’accord  3 – Plutôt d’accord  4 – Tout à fait d’accord | 1 – Pas du tout d’accord  2 – Plutôt pas d’accord  3 – Plutôt d’accord  4 – Tout à fait d’accord |
| Dépistage sur un holter ECG de suivi d'une hyperexcitabilité atriale superieure à 10000 ESA par 24h | 1 – Pas du tout d’accord  2 – Plutôt pas d’accord  3 – Plutôt d’accord  4 – Tout à fait d’accord | 1 – Pas du tout d’accord  2 – Plutôt pas d’accord  3 – Plutôt d’accord  4 – Tout à fait d’accord | 1 – Pas du tout d’accord  2 – Plutôt pas d’accord  3 – Plutôt d’accord  4 – Tout à fait d’accord | 1 – Pas du tout d’accord  2 – Plutôt pas d’accord  3 – Plutôt d’accord  4 – Tout à fait d’accord | 1 – Pas du tout d’accord  2 – Plutôt pas d’accord  3 – Plutôt d’accord  4 – Tout à fait d’accord |

*Définition de l'European staging*

*L’European staging est un outil utilisé pour stratifier les patients atteints d’amylose AL.*


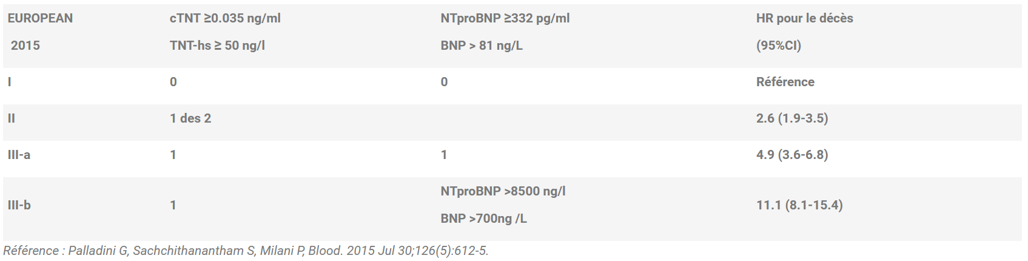


**FA ET FLUTTER (AL)**

**Indiquez votre degré d'accord dans les prises en charge de la fibrillation atriale proposées devant un patient amylose AL pour chacune des situations suivantes :**

|  | **Contrôle du rythme** | **Contrôle de la fréquence** |
| --- | --- | --- |
| Premier épisode de FA symptomatique | 1 – Pas du tout d’accord  2 – Plutôt pas d’accord  3 – Plutôt d’accord  4 – Tout à fait d’accord | 1 – Pas du tout d’accord  2 – Plutôt pas d’accord  3 – Plutôt d’accord  4 – Tout à fait d’accord |
| Premier épisode de FA asymptomatique | 1 – Pas du tout d’accord  2 – Plutôt pas d’accord  3 – Plutôt d’accord  4 – Tout à fait d’accord | 1 – Pas du tout d’accord  2 – Plutôt pas d’accord  3 – Plutôt d’accord  4 – Tout à fait d’accord |
| Après récidive post CEE symptomatique, et sous amiodarone | 1 – Pas du tout d’accord  2 – Plutôt pas d’accord  3 – Plutôt d’accord  4 – Tout à fait d’accord | 1 – Pas du tout d’accord  2 – Plutôt pas d’accord  3 – Plutôt d’accord  4 – Tout à fait d’accord |
| Après récidive post CEE asymptomatique, et sous amiodarone | 1 – Pas du tout d’accord  2 – Plutôt pas d’accord  3 – Plutôt d’accord  4 – Tout à fait d’accord | 1 – Pas du tout d’accord  2 – Plutôt pas d’accord  3 – Plutôt d’accord  4 – Tout à fait d’accord |

- **Indiquez votre degré d'accord :**

**Si le score de l'European staging est plus élevé, votre prise en charge sera plus agressive**
1 – Pas du tout d’accord 2 – Plutôt pas d’accord 3 – Plutôt d’accord 4 – Tout à fait d’accord

- **Vous avez choisi une stratégie de contrôle de la fréquence. Devant un patient amylose AL présentant une FA rapide avec FEVG préservée, indiquez votre degré d'accord avec chacune des thérapeutiques proposées, puis si le score European Staging est plus élevé :**

|  |  | **Si European staging plus elevé** |
| --- | --- | --- |
| Bétabloquants à doses progressives | 1 – Pas du tout d’accord  2 – Plutôt pas d’accord  3 – Plutôt d’accord  4 – Tout à fait d’accord | 1 – Pas du tout d’accord  2 – Plutôt pas d’accord  3 – Plutôt d’accord  4 – Tout à fait d’accord |
| Digoxine per os | 1 – Pas du tout d’accord  2 – Plutôt pas d’accord  3 – Plutôt d’accord  4 – Tout à fait d’accord | 1 – Pas du tout d’accord  2 – Plutôt pas d’accord  3 – Plutôt d’accord  4 – Tout à fait d’accord |
| Amiodarone à visée de ralentissement de la FC | 1 – Pas du tout d’accord  2 – Plutôt pas d’accord  3 – Plutôt d’accord  4 – Tout à fait d’accord | 1 – Pas du tout d’accord  2 – Plutôt pas d’accord  3 – Plutôt d’accord  4 – Tout à fait d’accord |
| Inhibiteurs calciques | 1 – Pas du tout d’accord  2 – Plutôt pas d’accord  3 – Plutôt d’accord  4 – Tout à fait d’accord | 1 – Pas du tout d’accord  2 – Plutôt pas d’accord  3 – Plutôt d’accord  4 – Tout à fait d’accord |
| Ablation NAV | 1 – Pas du tout d’accord  2 – Plutôt pas d’accord  3 – Plutôt d’accord  4 – Tout à fait d’accord | 1 – Pas du tout d’accord  2 – Plutôt pas d’accord  3 – Plutôt d’accord  4 – Tout à fait d’accord |

- **Vous avez choisi une stratégie de contrôle du rythme. Devant un patient amylose AL présentant une FA avec FEVG préservée, indiquez votre degré d'accord avec chacune des thérapeutiques proposées, puis si le score European Staging est plus élevé :**

|  |  | **Si European staging plus elevé** |
| --- | --- | --- |
| Ablation de FA | 1 – Pas du tout d’accord  2 – Plutôt pas d’accord  3 – Plutôt d’accord  4 – Tout à fait d’accord | 1 – Pas du tout d’accord  2 – Plutôt pas d’accord  3 – Plutôt d’accord  4 – Tout à fait d’accord |
| Amiodarone | 1 – Pas du tout d’accord  2 – Plutôt pas d’accord  3 – Plutôt d’accord  4 – Tout à fait d’accord | 1 – Pas du tout d’accord  2 – Plutôt pas d’accord  3 – Plutôt d’accord  4 – Tout à fait d’accord |
| Flécaine | 1 – Pas du tout d’accord  2 – Plutôt pas d’accord  3 – Plutôt d’accord  4 – Tout à fait d’accord | 1 – Pas du tout d’accord  2 – Plutôt pas d’accord  3 – Plutôt d’accord  4 – Tout à fait d’accord |
| Sotalol | 1 – Pas du tout d’accord  2 – Plutôt pas d’accord  3 – Plutôt d’accord  4 – Tout à fait d’accord | 1 – Pas du tout d’accord  2 – Plutôt pas d’accord  3 – Plutôt d’accord  4 – Tout à fait d’accord |
| CEE répétés | 1 – Pas du tout d’accord  2 – Plutôt pas d’accord  3 – Plutôt d’accord  4 – Tout à fait d’accord | 1 – Pas du tout d’accord  2 – Plutôt pas d’accord  3 – Plutôt d’accord  4 – Tout à fait d’accord |

- **Vous avez choisi une stratégie de cardioversion chez un patient amylose AL, anticoagulé de façon efficace. Indiquez votre degré d'accord avec la réalisation d'une imagerie systématique de l'auricule pour vérifier la présence d'un thrombus :**

1 – Pas du tout d’accord 2 – Plutôt pas d’accord 3 – Plutôt d’accord 4 – Tout à fait d’accord

- **Devant un patient amylose AL présentant un flutter atrial commun, indiquez votre degré d'accord avec la réalisation d'une ablation en première intention :**

1 – Pas du tout d’accord 2 – Plutôt pas d’accord 3 – Plutôt d’accord 4 – Tout à fait d’accord

**IMPLANTATION (AL)**

- **Indiquez votre degré d'accord vis-à-vis des prises en charge proposées. Devant un patient amylose AL asymptomatique pour chacune des situations suivantes :***(les différentes stratégies ne sont pas mutuellement exclusives, vous donnez votre avis pour chaque stratégie indépendamment)*

|  | **Pacemaker/Défibrillateur automatique implantable** | **Examen électrophysiologique** | **Enregistrement de longue durée** | **Rien** |
| --- | --- | --- | --- | --- |
| QRS fin et BAV I avec PR entre 200 et 250 ms | 1 – Pas du tout d’accord  2 – Plutôt pas d’accord  3 – Plutôt d’accord  4 – Tout à fait d’accord | 1 – Pas du tout d’accord  2 – Plutôt pas d’accord  3 – Plutôt d’accord  4 – Tout à fait d’accord | 1 – Pas du tout d’accord  2 – Plutôt pas d’accord  3 – Plutôt d’accord  4 – Tout à fait d’accord | 1 – Pas du tout d’accord  2 – Plutôt pas d’accord  3 – Plutôt d’accord  4 – Tout à fait d’accord |
| QRS fin et BAV I avec PR > 250 ms | 1 – Pas du tout d’accord  2 – Plutôt pas d’accord  3 – Plutôt d’accord  4 – Tout à fait d’accord | 1 – Pas du tout d’accord  2 – Plutôt pas d’accord  3 – Plutôt d’accord  4 – Tout à fait d’accord | 1 – Pas du tout d’accord  2 – Plutôt pas d’accord  3 – Plutôt d’accord  4 – Tout à fait d’accord | 1 – Pas du tout d’accord  2 – Plutôt pas d’accord  3 – Plutôt d’accord  4 – Tout à fait d’accord |
| QRS > 120 ms, bloc de branche droit, BAV I avec PR entre 200 et 250 ms | 1 – Pas du tout d’accord  2 – Plutôt pas d’accord  3 – Plutôt d’accord  4 – Tout à fait d’accord | 1 – Pas du tout d’accord  2 – Plutôt pas d’accord  3 – Plutôt d’accord  4 – Tout à fait d’accord | 1 – Pas du tout d’accord  2 – Plutôt pas d’accord  3 – Plutôt d’accord  4 – Tout à fait d’accord | 1 – Pas du tout d’accord  2 – Plutôt pas d’accord  3 – Plutôt d’accord  4 – Tout à fait d’accord |
| QRS > 120 ms, bloc de branche gauche, BAV I avec PR entre 200 et 250 ms | 1 – Pas du tout d’accord  2 – Plutôt pas d’accord  3 – Plutôt d’accord  4 – Tout à fait d’accord | 1 – Pas du tout d’accord  2 – Plutôt pas d’accord  3 – Plutôt d’accord  4 – Tout à fait d’accord | 1 – Pas du tout d’accord  2 – Plutôt pas d’accord  3 – Plutôt d’accord  4 – Tout à fait d’accord | 1 – Pas du tout d’accord  2 – Plutôt pas d’accord  3 – Plutôt d’accord  4 – Tout à fait d’accord |
| QRS > 120 ms, bloc de branche droit, BAV I avec PR > 250 ms | 1 – Pas du tout d’accord  2 – Plutôt pas d’accord  3 – Plutôt d’accord  4 – Tout à fait d’accord | 1 – Pas du tout d’accord  2 – Plutôt pas d’accord  3 – Plutôt d’accord  4 – Tout à fait d’accord | 1 – Pas du tout d’accord  2 – Plutôt pas d’accord  3 – Plutôt d’accord  4 – Tout à fait d’accord | 1 – Pas du tout d’accord  2 – Plutôt pas d’accord  3 – Plutôt d’accord  4 – Tout à fait d’accord |
| QRS > 120 ms, bloc de branche gauche, BAV I avec PR > 250 ms | 1 – Pas du tout d’accord  2 – Plutôt pas d’accord  3 – Plutôt d’accord  4 – Tout à fait d’accord | 1 – Pas du tout d’accord  2 – Plutôt pas d’accord  3 – Plutôt d’accord  4 – Tout à fait d’accord | 1 – Pas du tout d’accord  2 – Plutôt pas d’accord  3 – Plutôt d’accord  4 – Tout à fait d’accord | 1 – Pas du tout d’accord  2 – Plutôt pas d’accord  3 – Plutôt d’accord  4 – Tout à fait d’accord |
| Bloc trifasciculaire  Définition | 1 – Pas du tout d’accord  2 – Plutôt pas d’accord  3 – Plutôt d’accord  4 – Tout à fait d’accord | 1 – Pas du tout d’accord  2 – Plutôt pas d’accord  3 – Plutôt d’accord  4 – Tout à fait d’accord | 1 – Pas du tout d’accord  2 – Plutôt pas d’accord  3 – Plutôt d’accord  4 – Tout à fait d’accord | 1 – Pas du tout d’accord  2 – Plutôt pas d’accord  3 – Plutôt d’accord  4 – Tout à fait d’accord |
| Troubles conductifs évolutifs | 1 – Pas du tout d’accord  2 – Plutôt pas d’accord  3 – Plutôt d’accord  4 – Tout à fait d’accord | 1 – Pas du tout d’accord  2 – Plutôt pas d’accord  3 – Plutôt d’accord  4 – Tout à fait d’accord | 1 – Pas du tout d’accord  2 – Plutôt pas d’accord  3 – Plutôt d’accord  4 – Tout à fait d’accord | 1 – Pas du tout d’accord  2 – Plutôt pas d’accord  3 – Plutôt d’accord  4 – Tout à fait d’accord |
| Passage paroxystique en BAV II Mobitz 1 | 1 – Pas du tout d’accord  2 – Plutôt pas d’accord  3 – Plutôt d’accord  4 – Tout à fait d’accord | 1 – Pas du tout d’accord  2 – Plutôt pas d’accord  3 – Plutôt d’accord  4 – Tout à fait d’accord | 1 – Pas du tout d’accord  2 – Plutôt pas d’accord  3 – Plutôt d’accord  4 – Tout à fait d’accord | 1 – Pas du tout d’accord  2 – Plutôt pas d’accord  3 – Plutôt d’accord  4 – Tout à fait d’accord |
| Maladie Rythmique Atriale  Définition | 1 – Pas du tout d’accord  2 – Plutôt pas d’accord  3 – Plutôt d’accord  4 – Tout à fait d’accord | 1 – Pas du tout d’accord  2 – Plutôt pas d’accord  3 – Plutôt d’accord  4 – Tout à fait d’accord | 1 – Pas du tout d’accord  2 – Plutôt pas d’accord  3 – Plutôt d’accord  4 – Tout à fait d’accord | 1 – Pas du tout d’accord  2 – Plutôt pas d’accord  3 – Plutôt d’accord  4 – Tout à fait d’accord |
| Pause sinusale entre 3 et 6 sec. diurne | 1 – Pas du tout d’accord  2 – Plutôt pas d’accord  3 – Plutôt d’accord  4 – Tout à fait d’accord | 1 – Pas du tout d’accord  2 – Plutôt pas d’accord  3 – Plutôt d’accord  4 – Tout à fait d’accord | 1 – Pas du tout d’accord  2 – Plutôt pas d’accord  3 – Plutôt d’accord  4 – Tout à fait d’accord | 1 – Pas du tout d’accord  2 – Plutôt pas d’accord  3 – Plutôt d’accord  4 – Tout à fait d’accord |

*Définition de Bloc trifasciculaire*

*Un bloc trifasciculaire est un problème de conduction électrique du cœur, plus précisément des trois faisceaux des branches du faisceau qui transportent les signaux électriques du nœud auriculo-ventriculaire aux ventricules.*

*Définition de Maladie Rythmique Atriale*

*Une maladie rythmique atriale est un trouble du rythme cardiaque qui affecte les oreillettes du cœur. Cette maladie est caractérisée par une altération du tissu de l'oreillette, qui peut entraîner des anomalies du rythme cardiaque à fréquence variable. Les symptômes de la maladie rythmique atriale peuvent inclure des palpitations, des étourdissements, une fatigue, une douleur thoracique et une insuffisance cardiaque.*

- **Indiquez votre degré d'accord pour chaque critère. Face à un patient amylose AL ayant une indication de stimulation, les critères suivants influencent votre décision de mettre en place un défibrillateur :**

**La FEVG**
1 – Pas du tout d’accord 2 – Plutôt pas d’accord 3 – Plutôt d’accord 4 – Tout à fait d’accord

**Le Strain Global Longitudinal**
1 – Pas du tout d’accord 2 – Plutôt pas d’accord 3 – Plutôt d’accord 4 – Tout à fait d’accord

**Les TVNS**
1 – Pas du tout d’accord 2 – Plutôt pas d’accord 3 – Plutôt d’accord 4 – Tout à fait d’accord

- **Indiquez votre degré d'accord pour chaque critère. Face à un patient amylose AL n'ayant pas une indication de stimulation, les critères suivants influencent votre décision de mettre en place un défibrillateur :**

**La FEVG**
1 – Pas du tout d’accord 2 – Plutôt pas d’accord 3 – Plutôt d’accord 4 – Tout à fait d’accord

**Le Strain Global Longitudinal**
1 – Pas du tout d’accord 2 – Plutôt pas d’accord 3 – Plutôt d’accord 4 – Tout à fait d’accord

**Les TVNS**
1 – Pas du tout d’accord 2 – Plutôt pas d’accord 3 – Plutôt d’accord 4 – Tout à fait d’accord

- **Indiquez votre degré d'accord. Face à un patient amylose AL ayant une indication de stimulation ventriculaire :**

**La FEVG va influencer une décision de CRT**
1 – Pas du tout d’accord 2 – Plutôt pas d’accord 3 – Plutôt d’accord 4 – Tout à fait d’accord

**Un cut-off à 50% vous semble adapté**

1 – Pas du tout d’accord 2 – Plutôt pas d’accord 3 – Plutôt d’accord 4 – Tout à fait d’accord

**Le Strain Global Longitudinal va influencer une décision de CRT**
1 – Pas du tout d’accord 2 – Plutôt pas d’accord 3 – Plutôt d’accord 4 – Tout à fait d’accord

**Un cut-off à 14% vous semble adapté**

1 – Pas du tout d’accord 2 – Plutôt pas d’accord 3 – Plutôt d’accord 4 – Tout à fait d’accord

**Le pourcentage de stimulation attendu va influencer une décision de CRT**
1 – Pas du tout d’accord 2 – Plutôt pas d’accord 3 – Plutôt d’accord 4 – Tout à fait d’accord

**SATISFACTION
Comment avez-vous trouvé ce questionnaire ?**


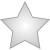
 
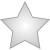
 
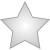
 
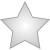
 
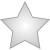


**Avez-vous des remarques / commentaires à faire sur le questionnaire auquel vous venez de répondre ?**

**CONCLUSION**

Ce questionnaire est maintenant terminé. Nous vous remercions pour votre participation.
